# Genetic and transcriptome analyses reveal the candidate genes and pathways involved in the inactive shade-avoidance response enabling high-density planting of soybean

**DOI:** 10.3389/fpls.2022.973643

**Published:** 2022-08-03

**Authors:** Jing Zhao, Xiaolei Shi, Lei Chen, Qiang Chen, Xuan Tian, Lijuan Ai, Hongtao Zhao, Chunyan Yang, Long Yan, Mengchen Zhang

**Affiliations:** ^1^Hebei Laboratory of Crop Genetics and Breeding, National Soybean Improvement Center Shijiazhuang Sub-Center, Huang-Huai-Hai Key Laboratory of Biology and Genetic Improvement of Soybean, Ministry of Agriculture and Rural Affairs, Institute of Cereal and Oil Crops, Hebei Academy of Agricultural and Forestry Sciences, Shijiazhuang, China; ^2^School of Life Sciences, Yantai University, Yantai, China; ^3^Key Laboratory of Molecular and Cellular Biology, Key Laboratory of Molecular and Cellular Biology of Ministry of Education, Hebei Collaboration Innovation Center for Cell Signaling, College of Life Science, Hebei Normal University, Shijiazhuang, China

**Keywords:** soybean, shade-avoidance syndrome, high-density planting, QTL- mapping, RNA-seq

## Abstract

High-density planting is a major way to improve crop yields. However, shade-avoidance syndrome (SAS) is a major factor limiting increased planting density. First Green Revolution addressed grass lodging problem by using dwarf/semi-dwarf genes. However, it is not suitable for soybean, which bear seeds on stalk and whose seed yield depends on plant height. Hence, mining shade-tolerant germplasms and elucidating the underlying mechanism could provide meaningful resources and information for high-yield breeding. Here, we report a high-plant density-tolerant soybean cultivar, JiDou 17, which exhibited an inactive SAS (iSAS) phenotype under high-plant density or low-light conditions at the seedling stage. A quantitative trait locus (QTL) mapping analysis using a recombinant inbred line (RIL) population showed that this iSAS phenotype is related to a major QTL, named *shade-avoidance response 1* (*qSAR1*), which was detected. The mapping region was narrowed by a haplotype analysis into a 554 kb interval harboring 44 genes, including 4 known to be key regulators of the SAS network and 4 with a variance response to low-light conditions between near isogenic line (NIL) stems. *Via* RNA-seq, we identified iSAS-specific genes based on one pair of near isogenic lines (NILs) and their parents. The iSAS-specific genes expressed in the stems were significantly enriched in the “proteasomal protein catabolic” process and the proteasome pathway, which were recently suggested to promote the shade-avoidance response by enhancing PIF7 stability. Most iSAS-specific proteasome-related genes were downregulated under low-light conditions. The expression of genes related to ABA, CK, and GA significantly varied between the low- and normal-light conditions. This finding is meaningful for the cloning of genes that harbor beneficial variation(s) conferring the iSAS phenotype fixed in domestication and breeding practice.

## Introduction

Currently, improving crop yields is extremely urgent (Evans, 2013; Ray et al., 2013). To solve the problem of feeding a growing population without expanding cultivated land area, high-density planting and intercropping have been widely used in modern practice (Brooker et al., 2015; Cao et al., 2022). However, the light environment within the canopy of these crops changes under both planting methods. Light serves as the basis of photosynthesis; photosynthesis is the most important process for obtaining organic products, provides basic materials for crop yield formation and is generally the principal factor affecting the yield of crops (Faralli and Lawson, 2020). It has been proven that the efficiency of light interception, the efficiency of converting intercepted light into biomass and the harvest index are three factors that are closely related to agricultural yields (Kubis and Bar-Even, 2019). Therefore, tapping into plant resources, such as tolerance to high planting density, and analyzing their underlying molecular mechanisms are key to improving crop yields.

In intercropping systems, the plants that grow slowly become shaded by taller plants. Moreover, under high plant density conditions, plants compete with each other for light. As a result of limited light availability, most plant species exhibit shade-avoidance syndrome (SAS) to escape from shade. Generally, the SAS response includes a series of characteristics, such as stem elongation, leaf hyponasty, reduced branching, phototropic orientation of the plant shoots toward gaps in the canopy, early flowering, and accelerated senescence (Ballaré, 1999; Bellaloui et al., 2012; Yang et al., 2014; Wu et al., 2017; Liu et al., 2021).

SAS is caused by changes in light conditions mainly driven by the presence of neighboring vegetation (Casal, 2013). The factors that trigger and control SAS responses mainly include phytochromes (phys), cryptochromes (crys), phototropins, and UV RESISTANCE LOCUS 8 (UVR8) (Ballaré, 1999; Franklin, 2008; Chen and Chory, 2011; Casal, 2012, 2013; Fraser et al., 2016; Ballaré and Pierik, 2017; Liu et al., 2021). In the SAS response, among the five PHYs (phyA-E), Phytochrome B (phyB), which senses a decrease in the red (R): far-red (FR) ratio, has been identified as the major photoreceptor in *Arabidopsis* (Franklin and Whitelam, 2005; Franklin, 2008; Keller et al., 2011; Casal, 2012; Liu et al., 2021; Lyu et al., 2021). While it has been proven that other PHYs act redundantly with phyB in controlling other developmental processes, phyD and phyE are involved in the flowering time and petiole elongation, while phyE is related to internode elongation (Martínez-García et al., 2014). Phytochrome exists in the following two photoreversible forms: an active FR light-absorbing (Pfr) form and an inactive R light-absorbing (Pr) form (Yamaguchi et al., 1999).

PHYTOCHROME-INTERACTING FACTORS (PIFs), including PIF3, PIF4, PIF5 and PIF7, constitute a group of regulators of SAS, and their activities can be repressed by active phyB (Franklin, 2008; Liu et al., 2021). Under shade conditions, PIF proteins bind and activate downstream targets involved in increased elongation growth, which are mostly cell wall-associated and auxin biosynthesis-related genes (Lorrain et al., 2008; Casal, 2013; Fraser et al., 2016; Liu et al., 2021). It has been proven that many small auxin up RNA (SAUR) genes are direct targets of PIF4 (Oh et al., 2012; Sun et al., 2016; Stortenbeker and Bemer, 2019). However, when overexpressed, SAURs promote cell elongation in *Arabidopsis* (Franklin et al., 2011; Chae et al., 2012; Spartz et al., 2012; Stamm and Kumar, 2013; Bemer et al., 2017; van Mourik et al., 2017).

Interestingly, a recent study showed that the shade-induced expression of *AUXIN RESPONSE FACTOR 18* (*ARF18*) can be inhibited by PIFs, which is followed by the repression of the auxin pathway (Jia et al., 2020). In addition, for SAS, the first identified candidate gene, *Early Flowering 3* (*ELF3*), has also been proven to play a role in phyB signaling, and its involvement is independent of its function in the circadian system (Reed et al., 2000; Botto and Smith, 2002; Yu et al., 2008; Thines and Harmon, 2010; Coluccio et al., 2011; Liu et al., 2011). In a recent study in *Arabidopsis*, PIF7-ELF3 interactions were suggested to play a role in hypocotyl elongation as a part of the shade response (Jiang et al., 2019).

In addition to the reduced R:FR ratio, when subjected to vegetational shading, plants also experience a reduction in blue light. Cryptochromes, which are types of photolyase-like blue light receptors, have been shown to participate in SAS (Franklin et al., 2011; Liu et al., 2011, 2021). By modulating hormone actions under shade conditions, *CRY1* and *CRY2* have been shown to be other key regulators involved in SAS in *Arabidopsis* (Franklin, 2008; Keuskamp et al., 2012; Casal, 2013; Pedmale et al., 2016). Moreover, it has been proven that both cry1 and cry2 interact with PIF4 and PIF5 (Ma et al., 2016; Pedmale et al., 2016).

Phototropins may play a role in shade avoidance by controlling phototropic growth in response to gradients of blue light (Galen et al., 2004; Casal, 2013; Fraser et al., 2016; Pierik and Ballare, 2021). For phototropism, PHOTOTROPIN 1 (PHOT1), and PHOT2 are photoreceptors (Christie et al., 1998; Briggs and Christie, 2002), and PHOT1 has been proven to interact with NON-PHOTOTROPIC HYPOCOTYL 3 (NPH3) (Motchoulski and Liscum, 1999). PHOT1 has also been suggested to function genetically upstream of PIF4 and PIF5, whose repression of auxin signaling plays a critical role in phototropism (Sun et al., 2013).

In plants, SAS caused by high planting density is accompanied by many unfavorable phenotypic characteristics, such as lodging and decreased photosynthesis, which result in yield reductions (Liu et al., 2021). Therefore, breeding and cultivating plant varieties that are tolerant to high-density conditions are important strategies for achieving crop yield breakthroughs in the future (Wang and Wang, 2021).

Soybean [*Glycine max* (L.) Merr.], which is native to China, is among the major legume crop species worldwide and serves as an important source of protein and oil (Ainsworth et al., 2012); thus, soybean is among the most economically important crop species (Liu et al., 2020). Previous research indicated that a rate of increase in soybean production of 2.4% per year is needed by 2050 to meet projected demands (Ray et al., 2013). Hence, achieving such a goal is an enormous challenge. For soybean yield improvements, high-density planting methods similar to those used for cereal crops have been practiced (Lyu et al., 2021; Mu et al., 2022). However, for soybean, it has been proven that plant density should be within an optimum range; increased plant density beyond this range could lead to reduced yields due to interplant competition (Egli, 1988). In addition, taller plants within thinner and weaker stems caused by the SAS response could have reduced lodging resistance. Many measures have been taken to solve these problems. Lyu et al. (2021) showed that *GmCRY1s* could modulate the gibberellin (GA) metabolic pathway in the regulation of low blue light (LBL)-induced SAS in soybean, indicating that manipulating genes downstream of *GmCRY1s* could trigger further yield improvement in soybean. *GmMYB14*, which is involved in the plant architecture through brassinosteroid (BR) signaling pathways, has also been studied in attempts to increase soybean yields (Chen et al., 2021). *GmBICs*, which regulate stem elongation, also have potential in controlling the plant architecture for yield improvement in soybean (Mu et al., 2022). A recent study reported that the plant architecture could be improved by regulating the leaf petiole angle *via* the auxin efflux transporter *PINFORMED 1* (*GmPIN1*) in soybean (Zhang et al., 2022).

As SAS is a major limiting factor in high-density planting, exploring shade-tolerant germplasm and elucidating the underlying mechanisms could provide meaningful resources and genetic information for breeding high-yielding plants driven by high-density planting. Previously, we bred a high-plant density-tolerant cultivar, JiDou 17 (JD17), which has a wide range of regional adaptability and high yield performance (Zhao et al., 2019). However, the underlying mechanism of its phenotype is still unclear. Here, we combined genetic and transcriptomic analyses to mine the candidate genes and elucidate the molecular mechanism underlying the high-plant density tolerance phenotype of JD17.

## Materials and methods

### Plant materials and growth conditions

Two soybean cultivars, JD17 and JiDou 12 (JD12, with a strong SAS phenotype), were crossed to develop a recombinant inbred line (RIL) population, named 1712, *via* single-seed descent (SSD). The field trial was carried out at the Dishang Experimental Farm (E114.48°, N38.03°) of the Institute of Cereal and Oil Crops, Hebei Academy of Agricultural and Forestry Sciences, Shijiazhuang city, Hebei Province, China. In total, 199 F_9_ RILs were derived to construct a genetic linkage map and detect QTLs associated with the shade-avoidance response (SAR) trait, termed *qSAR1*. Based on phenotypic observation, one pair of near isogeneic lines (NILs), NIL-5 and NIL-33, related to *qSAR1* were selected from an F_10_ residual heterozygous line (RHL), of which, the NIL-5 inherited the phenotype in plant height of JD17, and has an inactive SAS under low-light condition, while the NIL-33 inherited of JD12 and has a strong SAS under the same light condition. For the QTL detection, a growth chamber labeling experiment was conducted. The phenotypes of the plants were observed and analyzed under a 16 h light/8 h dark photoperiod at 28°C in an artificial climate chamber, and the photosynthetic photon flux density (PPFD) was ∼660 μmol m^–2^ s^–1^ (50% of normal-light). The plant height was measured at 8 DAP, 13 DAP, 18 DAP, 23 DAP, 28 DAP, and 33 DAP.

### Microscopy analysis

The stem samples of JD12 and JD17 were collected for scanning electron microscopy (SEM) analysis. The plants growing in normal- and low-light (50% the normal-light condition) in a growth chamber at 8 DAP were used for samples collection.

### Genetic linkage map construction

A genetic linkage map (Supplementary Figure 1) was constructed using 1970 SNP markers from genotyping-by-sequencing (GBS). Genomic DNA was extracted from parents and RILs using a Plant Genomic DNA Kit (TIANGEN, Beijing, China) following the manufacturer’s protocol. SNP genotyping was conducted using a GBS approach. The GBS library was constructed as previously described (Cheng et al., 2015). Paired-end (PE) sequencing was carried out on the selected tags using an Illumina 2500 platform (Illumina, United States) by the staff at the Novogene Bioinformatics Institute, Beijing, China.

Burrows–Wheeler Aligner (BWA), SAMtools and a custom Perl script were used to identify the SNPs in the RIL population (Zhou et al., 2016). The software tool ANNOVAR (Wang et al., 2010) was used to align and annotate the SNPs or insertions-deletions (InDels) based on gff3 files from the soybean genome annotation obtained from the Phytozome database.

The map covers all 20 chromosomes, and the lengths of the individual linkage groups range from 80.86 to 191.43 cM. The total length of the map was 2534.4 cM, with an average marker density of 1.27 cM per marker and 62–136 markers per linkage group.

### QTL analysis

The quantitative trait locus (QTL) test was carried out using the R packages qtl and qtl2 (Broman et al., 2003, 2019) according to the user guide.^1^

### RNA isolation and quantitative real-time PCR

For genes expression validation, the total RNA was isolated from different soybean lines and tissues at 8 DAP seedlings. An RNA Prep Pure Plant Kit (TIANGEN DP-432) was used to extract the total RNA. Quantitative real-time PCR (qRT–PCR) was performed on a CFX96 Real-Time PCR Detection System (Bio-Rad) in conjunction with TB Green Fast qPCR Mix (TaKaRa, RR430A), with three biological replications. For the qRT–PCR analysis, the soybean *CYP2* (*Glyma.12G024700.1*) gene (Funatsuki et al., 2014) served as an internal control, and the relative gene expression was calculated using the 2^–Δ Δ^ *^Ct^* method. The primers used are listed in Supplementary Table 1.

### RNA-seq and bioinformatics analysis

Forty-eight paired-end (PE) RNA-seq libraries were generated from three biological replicates of each JD17-normal light-stem (NS17), JD17-normal light-root (NR17), JD17-low light-stem (LS17), JD17-normal light-root (LR17), JD12-normal light-stem (NS12), JD12-normal light-root (NR12), JD12-low light-stem (LS12), JD12-normal light-root (LR12), NL5-normal light-stem (NS5), NL5-normal light-root (NR5), NL5-low light-stem (LS5), NL5-normal light-root (LR5), NL33-normal light-stem (NS33), NL33-normal light-root (NR33), NL5-low light-stem (LS33), and NL33-normal light-root (LR33) data set (Figure 4A). The libraries were sequenced on an Illumina NovaSeq 6000 sequencing platform. Approximately 6 Gb of clean data were generated for each library.

**FIGURE 1:**
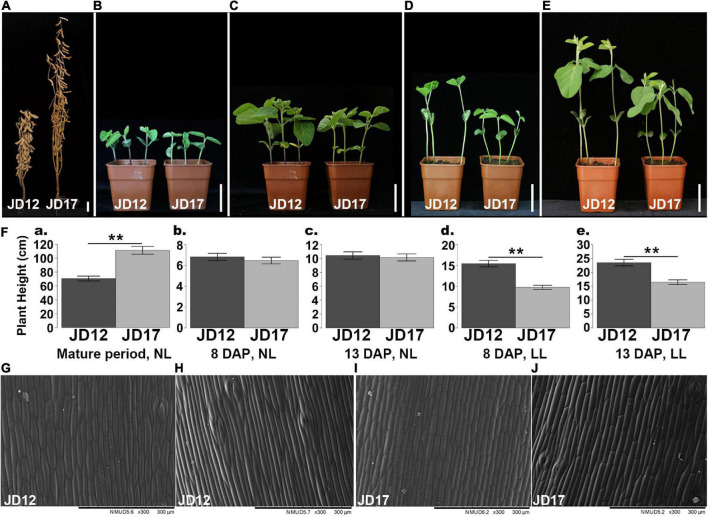
Phenotypes of JD17 compared with JD12. **(A)** Mature plants grown under field conditions; **(B)** plants at 8 and 13 **(C)** DAP grown in normal light; **(D)** plants at 8 and 13 **(E)** DAP grown in low-light in a growth chamber; **(F)** plant height comparisons of JD12 and JD17 grown in normal-light (NL) and low-light (LL) in a growth chamber at **(a)** mature period, **(b,d)** 8 DAP and **(c,e)**13DAP (means ± SD, *n* = 12); **(G)** hypocotyl epidermal cells of JD12 as shown *via* SEM at 8 DAP grown in normal light and **(H)** low-light in a growth chamber; **(I)** hypocotyl epidermal cells of JD17 as shown *via* SEM at 8 DAP grown in normal light and **(J)** low light in a growth chamber. The Student’s t-test was used for the comparisons: **P ≤ 0.01. Scale bars: **(A–E)** 5 cm; **(H–J)** 300 μm.

**FIGURE 2:**
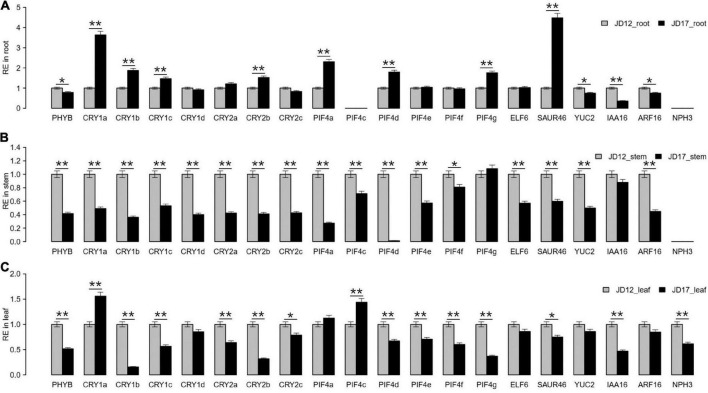
Expression patterns of SAS-related genes in JD17 and JD12. **(A)** Relative expression (RE) value in root, **(B)** stem and **(C)** leaf tissues. *CYP2* was used as an internal control in the qRT–PCR analyses. Student’s *t*-test was used for the statistical analysis: *P ≤ 0.05; **P ≤ 0.01. The values are the means ± SD (*n* = 3). The corresponding gene IDs and primer information are listed in Supplementary Table 1.

**FIGURE 3:**
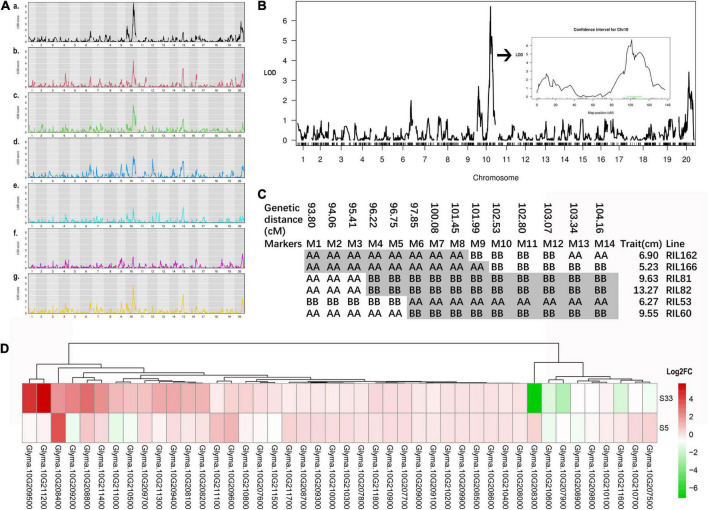
QTL detection of qSAR1. **(A)** Detection of the exact stage at which qSAR1 functions, **a-f** correspond to 8 DAP, 13 DAP, 18 DAP, 23 DAP, 28 DAP, and 33 DAP. **(B)** Details of the qSAR1-ch10 QTL at the most significant stage—8 DAP. **(C)** Haplotype analysis near the qSAR1-ch10 QTL peak. M1-M14 represent 14 SNP markers, which are listed in Supplementary Table 2. The gray color in each line indicates the putative chromosome regions in which qSAR1 may be located, which was predicted by the trait value of the line listed on the right. **(D)** Expression characteristics of 44 candidate genes in response to low light in near-isogenic lines. The log2FC showed log2 value of gene expression fold change under a 16 h light/8 h dark photoperiod at 28°C in an artificial climate chamber, and the photosynthetic photon flux density (PPFD) was ∼660 μmol m^– 2^ s^– 1^ (50% of normal light).

**FIGURE 4:**
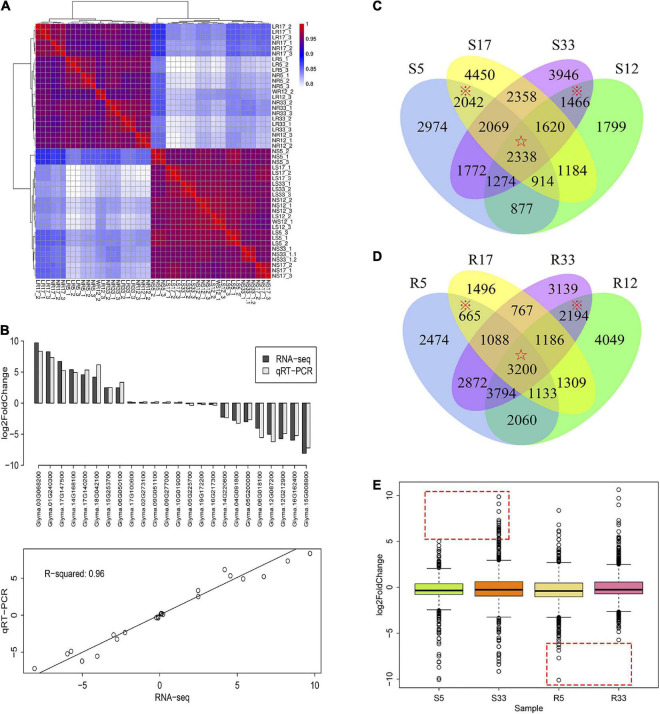
Transcriptome expression patterns of NIL-5, NIL-33, JD17, and JD12. **(A)** The expression pattern correlation of the 48 RNA-seq libraries shown by a Spearman heatmap. The figure was drawn in R language using the pheatmap package. **(B)** Validation of the RNA-Seq results by qRT–PCR: The upper figure shows the average expression value of the test genes, and the lower figure shows the correlation degree of the two methods. **(C)** Venn diagram showing the intersecting relationships of the expressed gene sets in the stem tissues of NIL-5 (R5), NIL-33 (R33), JD17 (R17), and JD12 (R12) at 8 DAP. **(D)** Venn diagram showing the intersecting relationships of the expressed gene sets in the root tissues of NIL-5 (R5), NIL-33 (R33), JD17 (R17), and JD12 (R12) at 8 DAP. **(E)** Boxplots showing the consensus genes [which are indicated under the star symbols in **(C,D)**]. Log2(FC) distribution patterns of NIL-5 and NIL-33.

Quality control and cleaning of the sequenced raw data were performed *via* fastQC and Trimmomatic software, respectively, in the Linux system of our local server. Then, the reads were mapped onto the W82 soybean reference genome (V2.1) using HISAT2 software. The sam files were converted to bam files and sorted using SAMtools. The transcripts were assembled and merged using StringTie software. The gene expression levels [transcripts per kilobase of exon model per million mapped reads (TPM)] were measured using Ballgown software. The differentially expressed genes (DEGs) were determined using the R language package DESeq2. All other statistical analyses and figures were generated and constructed in the R language environment. A gene cluster analysis was carried out using the TCseq package in R language.

## Results

### JiDou 17 presents an inactive shade-avoidance syndrome response at the seedling stage under high-density planting and low-light conditions

As the main phenotypic feature of SAS in plants under shade conditions, hypocotyl elongation is promoted at the seedling stage (Casal, 2012). However, the thinner and fragile stems of plants resulting from SAS are considered unfavorable factors for high-plant density tolerance, lodging resistance and yield.

Fortunately, we found that one of our previously bred varieties, JD17, exhibits an inactive SAS (iSAS) response at the seedling stage under high-density planting conditions. Throughout decades of production experiments, JD17 was characterized as a soybean cultivar that presented strong lodging resistance (Zhao et al., 2015) and was identified as being shade tolerant (Zhao et al., 2019). Interestingly, compared to another elite cultivar, JD12, JD17 exhibits an iSAS response (which was manifested as a significantly lower plant height and hypocotyl length compared with those of JD12) under high-density planting conditions and low-light conditions (∼50% of normal light intensity) in the seedling stage (Figures 1D,E,Fd,e), but JD17 is significantly taller than JD12 at the mature stage (Figures 1A,Fa). However, there was no obvious difference in plant height or hypocotyl length at the seedling stage under normal light conditions (Figures 1B,C,Fb,c). Taken together, these observations indicate that JD17 is insensitive to changes in light intensity. As the significant plant height various in JD17 and JD12 under different light condition, we speculated that there may be differences in elongation and/or division of the stem cells between these two materials. Scanning electron microscopy (SEM) observations revealed that the elongation rate of JD17 hypocotyl cells under low-light was significantly lower than that of JD12 cells under the same conditions (Figures 1G–J).

### Expression patterns of shade-avoidance syndrome-related genes widely varied between JiDou 17 and JiDou 12

To investigate whether the SAS phenotypic differences between JD17 and JD12 are related to SAS network genes (Supplementary Table 1), using quantitative real-time PCR (qRT–PCR), we measured the expression patterns of 20 SAS-related genes in the root, stem and leaf tissues of the two cultivars 8 days after planting (DAP) (our previous study (data not shown) showed that the plant height differed most significantly at 8 DAP). The results showed that the gene expression patterns in the stem tissue varied most significantly; 17 of the 20 target genes (excluding *PIF4g, IAA16*, and *NPH3*) were significantly downregulated in JD17 compared to JD12 (Figure 2B). In contrast, approximately half of the 20 genes were significantly upregulated in the root tissue of JD17 (Figure 2A). In the leaf tissue, 15 of the 20 genes’ expression levels significantly differed between JD12 and JD17, including 2 genes, *CRY1a*, and *PIF4c*, that were upregulated and 13 genes that were downregulated (Figure 2C). Notably, the expression of the *NPH3* gene was undetectable in both the root and stem tissues (Figures 2A,B). Moreover, the *PIF4c* gene was not expressed in the roots (Figure 2A) in either JD12 or JD17, whereas all three genes were expressed in the leaf tissue (Figure 2C); *PIF4c* was downregulated in JD17 stems (Figure 2B) but upregulated in JD17 leaves (Figure 2C), whereas *NPH3* was downregulated in JD17 leaves (Figure 2C).

### QTL mapping of shade-avoidance response 1 and candidate gene analysis

Based on the above analysis, we speculated that there is a gene/QTL in JD17 that leads to an inactive shade-avoidance response; we defined this QTL as *shade-avoidance response 1* (*qSAR1*). To identify the *qSAR1* QTL(s) in JD17 that drive the excellent iSAS trait, we constructed an RIL population named 1712 derived from the hybridization of JD17 × JD12. A genetic map containing 1970 genotyping-by-sequencing (GBS) single-nucleotide polymorphisms (SNPs) was developed to identify the iSAS QTL *qSAR1* (Supplementary Figure 1). To determine the exact stage at which the genes/QTLs functioned, we detected iSAS QTLs at six stages, i.e., 8 DAP (Figure 3Aa), 13 DAP (Figure 3Ab), 18 DAP (Figure 3Ac), 23 DAP (Figure 3Ad), 28 DAP (Figure 3Ae), and 33 DAP (Figure 3Af), and the average across these six stages (Figure 3Ag). The results revealed a conserved QTL on chromosome 10, termed qSAR1-chr10, in all 7 data sets (Figures 3Aa–g). However, the logarithm of the odds (LOD) value of the qSAR1-chr10 QTL was the most significant at 8 DAP (Figure 3Aa) and tended to decrease with the growth stage (Figures 3Ab–f). In addition to the major QTL located on chromosome 10, two minor QTLs were detected on chromosome 15 and chromosome 20. However, the LOD values of these two QTLs were too low to be further considered in our subsequent analysis.

To further explore the candidate genes for the major QTL qSAR1-chr10 at 8 DAP (Figure 3B), we further analyzed the haplotype pattern of the QTL peak region of six key F_2_ recombinant-derived lines. The haplotype pattern indicated that the qSAR1-chr10 QTL was located in a 554 kb interval region between markers 6 (M6, Gm10_43899046) and M8 (Gm10_44452705), ranging from 3.6 cM (Figure 3C and Supplementary Table 2). There were 44 annotated genes located in the putative region based on the information within the Phytozome database^2^ (Supplementary Table 3). Notably, 4 of the 44 candidate genes, i.e., *Glyma10G209600* (*ELF6*), *Glyma.10g209700* (*SAUR46*), *Glyma.10g210200* (*NPH3*), and *Glyma.10g210600* (*ARF16*), are potential key regulators related to SAS mentioned above in our qRT–PCR analysis results. The qRT–PCR results showed that the expression level of *SAUR46* was significantly upregulated in JD17 root tissue compared with JD12 root tissue (Figure 2A). In contrast, *SAUR46* was downregulated in both the stems and leaves of JD17 (Figures 2B,C). *ARF16* was significantly downregulated only in JD17 stems compared with JD12 stems (Figure 2B). *NPH3* was detectable only in leaf tissue and was significantly downregulated in JD17 compared with JD12 (Figure 2).

Then, used RNA sequencing (RNA-seq) method, we further detected the expression response of the 44 candidate genes in the stem tissues of the NIL materials (S5 and S33) under low-light conditions (half the normal light condition) compared to that under normal light conditions. The results showed that the response intensity of several genes under low-light significantly differed between the near-isogenic lines. The expression levels of two genes, *Glyma.10G209500* and *Glyma.10G211200*, were dramatically upregulated in S33 under low-light conditions compared with S5 (Figure 3D). In contrast, the expression level of the *Glyma.10G208400* gene was dramatically upregulated in S5. Another gene, *Glyma.10G208300*, was significantly downregulated in S33 but upregulated in S5.

### Global transcriptome profiling of the weak and strong shade-avoidance syndrome phenotypes

To preliminarily elucidate the molecular basis underlying the inactive/weak SAS phenotype of JD17, a set of RNA-seq experiments was devised. In addition to the two materials JD17 and JD12, one pair of near isogenic lines (NILs), i.e., NIL-5 and NIL-33, which were generated from an F_10_ RHL of 1712 and exhibited significant differences in SAS characteristics, were used for the analysis. Based on the significant variance in SAS-related genes expressed in the stem and root organs between JD17 and JD12, we further investigated the variance in the stem and root transcriptome profiles between normal-light conditions and low-light conditions of the four materials at 8 DAP. The detailed information is described in “Materials and Methods” section.

In total, we obtained 288 Gb of clean data from 48 RNA-seq libraries, and 90% of the clean reads in each library reached the necessary Q30 quality. Then, the expression levels of 24 randomly selected genes were validated *via* qRT–PCR. Eight of the 24 genes were randomly selected from among the upregulated genes, 8 were randomly selected from among the downregulated genes, and 8 were randomly selected from the non-significantly different gene sets. The results demonstrated that the results of the two methods presented good consistency, with an R square value of 0.96 (Figure 4B). The Spearman’s correlation coefficient analysis of the gene expression values revealed that the expression pattern of each sample could represent its tissue type and light condition (Supplementary Figure 2). This finding revealed that the RNA-seq data sets could be used for further analysis.

The differentially expressed gene (DEG) analysis demonstrated that the log2(fold change) (log2FC) of most DEG expression variances ranged from –5 to 5 in all 8 types of tissues (Supplementary Figure 2A). The expression level of a certain number of DEGs also changed dramatically; the log2FC of these expression changes was larger than 5 or lower than –5, with a maximum of 10 and a minimum of –10 (Supplementary Figures 2B,C).

To identify the sets of expressed genes specifically related to the iSAS and strong SAS response, genes with *p*-values < 0.05 were identified as being expressed and are shown in Venn diagrams (Figures 4C,D). Those genes expressed in both JD17 and NIL-5 and not expressed in JD12 or NIL-33 were identified as specific iSAS genes; 2,042 and 665 of these genes are shown under asterisks in the stem and root Venn diagrams, respectively, in Figures 4C,D. Those genes expressed in both JD12 and NIL-33 but in neither JD17 nor NIL-5 were identified as strong SAS-specific genes (sSAS); 1,466 and 2,194 of these genes are shown under asterisks in the stem and root Venn diagrams, respectively, shown in Figures 4C,D. Then, the expression log2(FC) values in the 4 data sets, i.e., the iSAS-stem (2,042 genes), iSAS-root (665 genes), sSAS-stem (1,466 genes), and sSAS-root (2,194) data sets, were extracted from the NIL samples, i.e., NIL-5-stem (S5), NIL-5-root (R5), NIL-33-stem (S33), and NIL-33-root (R33), respectively, and are shown in boxplots. The expression patterns demonstrated that the sSAS-stem genes were upregulated more strongly than the iSAS-stem genes, and 26 genes had log2(FC) values greater than 5 (Figure 4E and Supplementary Table 4). In contrast, the iSAS-root genes were downregulated more strongly than the sSAS-root genes, and 16 genes had log2(FC) values lower than –5 (Figure 4E and Supplementary Table 5). Similarly, the expression levels of the consensus genes, which are indicated under the star symbols, were downregulated more strongly in NIL-5-stem (S5) than NIL-33-stem (S33) (Figure 4E). Notably, a few genes associated with cell development, i.e., *Glyma.09G188800* (a glycine-rich cell wall structural protein), *Glyma.01G240300* (a glycine-rich cell wall structural protein) and *Glyma.12G096900* (cellulose synthase-like protein H1), were dramatically upregulated in S33.

**FIGURE 5:**
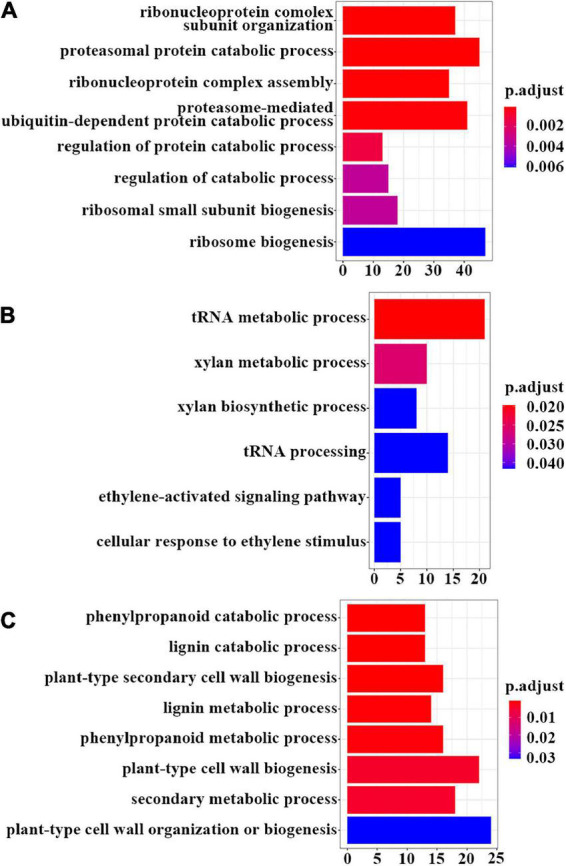
GO enrichment results of sets of specifically expressed genes shown as bar plots. **(A)** Bar plot of the GO enrichment results of the genes specifically expressed in the stem tissues of the iSAS samples (iSAS-stem). **(B)** Bar plot of the GO enrichment results of the genes specifically expressed in the stems of the strong-SAS sample (sSAS-stem) and **(C)** the roots of the strong-SAS samples (sSAS-root).

**FIGURE 6:**
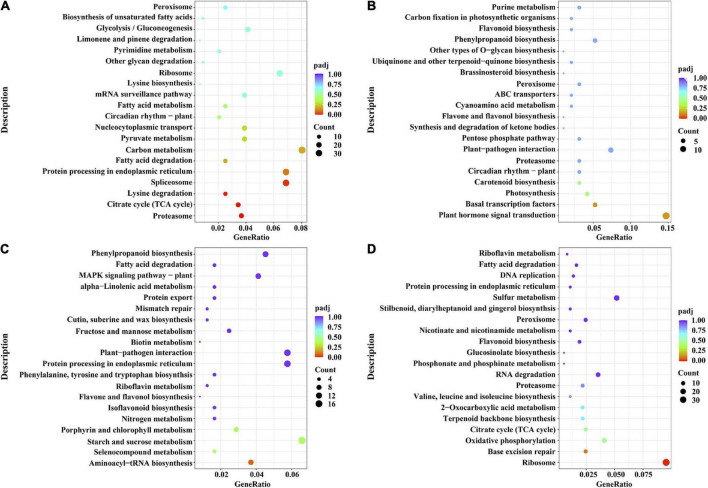
KEGG enrichment results of the sets of genes specifically expressed shown as dot plots. **(A)** Dot plot of the KEGG enrichment results of the genes specifically expressed in the stems of the iSAS samples (iSAS-stem) and **(B)** the roots of the iSAS samples (iSAS-root); **(C)** dot plot of the KEGG enrichment results of the genes specifically expressed in the stems of the strong-SAS samples (sSAS-stem) and **(D)** the roots of the strong-SAS samples (sSAS-root).

To further determine which biological processes the iSAS-specific gene sets were enriched in and what detailed metabolic pathways they participated in, Gene Ontology (GO) and Kyoto Encyclopedia of Genes and Genomes (KEGG) enrichment analyses were conducted. The results showed that the iSAS-stem gene set was enriched in the GO biological progress terms “ribonucleoprotein complex subunit organization,” “proteasomal protein catabolic process,” “ribonucleoprotein complex assembly,” “proteasome-mediated ubiquitin-dependent protein catabolic process,” “regulation of protein catabolic process,” etc. (Figure 5A and Supplementary Figure 3A). This finding is consistent with the KEGG enrichment results in which the genes were mainly enriched in the pathways “proteasome,” “citrate cycle (TCA cycle),” “lysine degradation,” and “spliceosome” (Figure 6A and Supplementary Figure 4A). Interestingly, the iSAS-root gene set was not enriched in any GO biological process terms. While, these genes were enriched in the KEGG pathways “plant hormone signal transduction” and “basal transcription factors” (Figure 6B and Supplementary Figure 4B). The sSAS-stem gene set was enriched in the GO biological processes “tRNA metabolic process” and “xylan metabolic process” (Figure 5B and Supplementary Figure 3B). Consistently, these genes were enriched in the KEGG pathways “aminoacyl-tRNA biosynthesis,” “selenocompound metabolism,” “starch and sucrose metabolism” and “porphyrin and chlorophyll metabolism” (Figure 6C and Supplementary Figure 4C). The sSAS-root gene sets were enriched in the GO biological progress terms “phenylpropanoid catabolic process,” “lignin catabolic process,” “plant-type secondary cell wall biogenesis,” “lignin metabolic process,” “phenylpropanoid metabolic process,” “plant-type cell wall biogenesis,” and “secondary metabolic process” (Figure 5C and Supplementary Figure 3C). However, the KEGG pathways “ribosome,” “base excision repair,” “oxidative phosphorylation,” “citrate cycle (TCA cycle),” etc., were enriched (Figure 6D and Supplementary Figure 4D).

**FIGURE 7:**
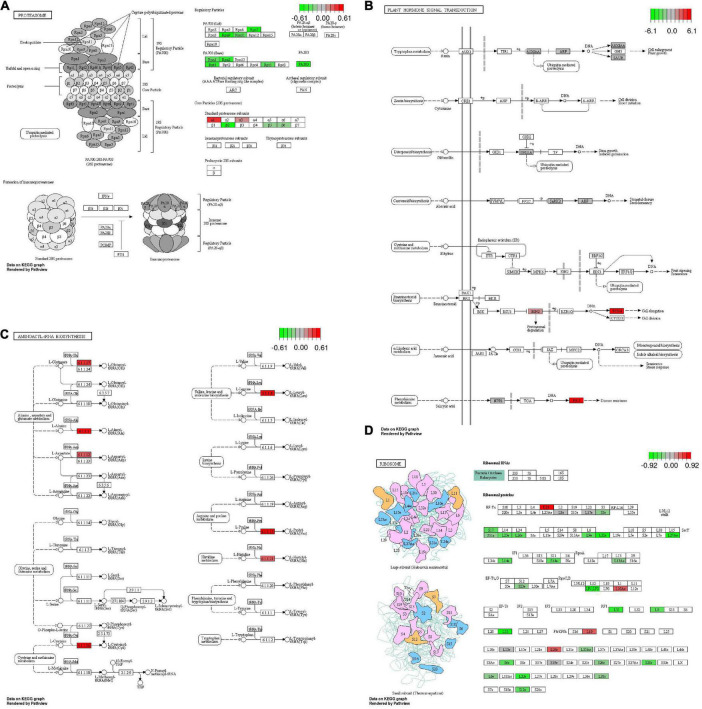
Mapping of the most significantly enriched KEGG pathways of the four sets of specifically expressed genes. **(A)** Expression patterns of the genes that are specifically expressed the stems of the iSAS samples (iSAS-stem) that are involved in the significantly enriched pathway “proteasome.” **(B)** Expression patterns of the genes that are specifically expressed in the roots of the iSAS samples (iSAS-root) that are involved in the significantly enriched pathway “plant hormone transduction.” **(C)** Expression patterns of the genes that are specifically expressed in the stems of the strong-SAS samples (sSAS-stem) and that are involved in the significantly enriched pathway “aminoacyl-tRNA biosynthesis.” **(D)** Expression patterns of the genes that are specifically expressed in the roots of the strong-SAS samples (sSAS-root) and that are involved in the significantly enriched pathway “ribosome.”

The genes enriched in the KEGG pathways in the four gene sets were further mapped to the corresponding pathways (Figure 7). The results demonstrated that for the most significantly enriched pathway (“proteasome”) in the iSAS-stem gene set, most enriched genes were downregulated (Figure 7A). However, in the iSAS-root gene set, two of the three enriched genes, *BIN2* and *TCH4*, were sharply upregulated in the BR signal transduction pathway, and *PR-1* was dramatically upregulated in the salicylic acid (SA) signal transduction pathway (Figure 7B). Similar to the iSAS-root genes, all of the most enriched genes in the sSAS gene set involved in the “aminoacyl-tRNA biosynthesis” pathway were upregulated (Figure 7C). Notably, in the sSAS-root gene set, most enriched genes were downregulated in the most significantly enriched pathway (“ribosome”) (Figure 7D).

### Expression patterns of consensus genes and key clusters of genes related to the shade-avoidance syndrome response

**FIGURE 8:**
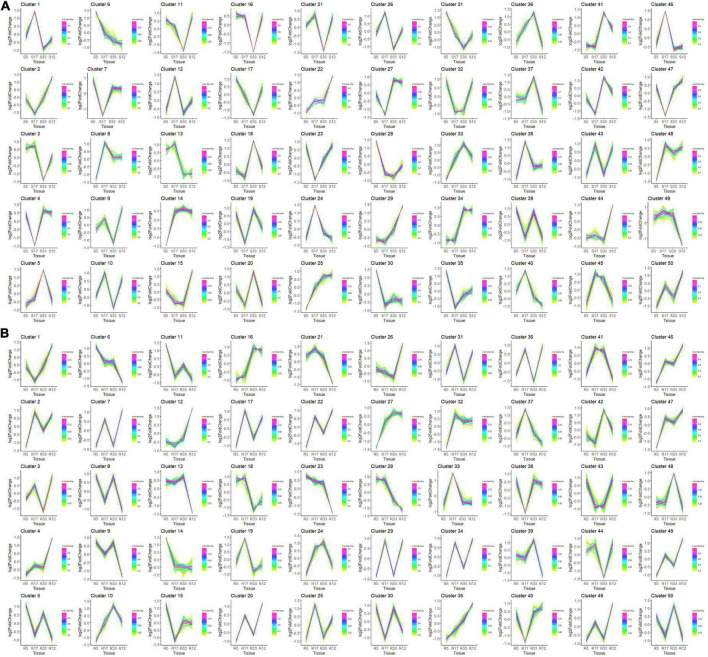
Gene cluster analysis results of the consensus gene expression patterns in the stem and root tissues of four samples. **(A)** Expression patterns of the top 50 gene clusters in the stem tissue of NIL-5 (S5), NIL-33 (S33), JD17 (S17), and JD12 (S12) at 8 DAP; **(B)** expression patterns of the top 50 gene clusters in the root tissue of NIL-5 (R5), NIL-33 (R33), JD17 (R17), and JD12 (R12) at 8 DAP.

To identify the clusters of genes corresponding to the SAS phenotype based on the gene expression patterns, a clustering analysis and visualization of the consensus genes shown under the star symbols in Figures 4C,D was conducted in the R language environment. The results demonstrated that although all consensus genes were expressed in the four samples, the expression trends and degree of response to light stimulation varied in both the stem and root tissues (Figure 8). Fortunately, there were four gene clusters with expression patterns corresponding to the SAS phenotype, i.e., cluster 13 (these genes were upregulated in both the S5 and S17 stem tissues and downregulated in both the S33 and S12 stem tissues), cluster 34 (these genes were downregulated in both the S5 and S17 stem tissues and upregulated in both the S33 and S12 tissues) (Figure 8A), cluster 16 (these genes were downregulated in both the S5 and S17 root tissues and upregulated in both the S33 and S12 root tissues) and cluster 18 (these genes were upregulated in both the S5 and S17 root tissues and downregulated in both the S33 and S12 root tissues) (Figure 8B).

The gene annotations of the four clusters showed that many genes were involved in cell wall biogenesis, light stimulus, phloem or xylem histogenesis and development, and many genes were involved in the regulation of transcription (Supplementary Table 6).

### Variation in the expression patterns of hormone-related genes associated with the inactive shade-avoidance syndrome and strong shade-avoidance syndrome phenotypes

**FIGURE 9:**
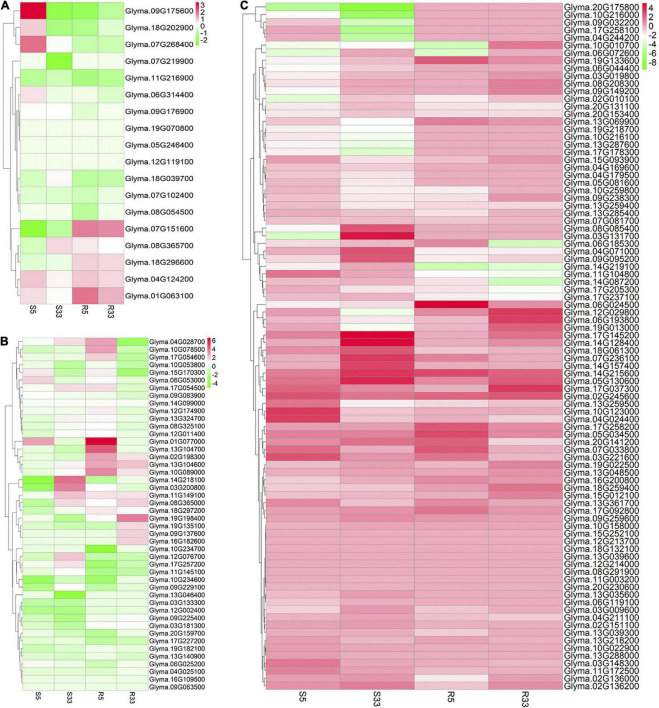
Variation in the expression patterns of plant hormone-related genes according to the RNA-seq transcriptome data. **(A)** Variation in the expression patterns of ABA-related genes, **(B)** CK-related genes and **(C)** GA-related genes. The bars in each map indicate the gene expression level (log2FC) in NILs grown under low-light conditions compared to those grown under high-light conditions.

Many previous studies have reported that plant hormones play key roles in SAS phenotype formation (Liu et al., 2021). In general, GAs and indole-3-acetic acid (IAA) promote the elongation of plant cells, cytokinins (CKs) are involved in cell division, and abscisic acid (ABA) and ethylene inhibit cell elongation. To investigate the mode of action of plant hormones in the SAS response, we further evaluated the variation in the expression patterns of genes involved in five plant hormones, i.e., IAA, GA, CK, ABA, and ethylene, according to the RNA-seq data. The results showed that one-third (6/18) of the ABA pathway-related genes were significantly upregulated in NIL-5-stem under low-light conditions compared with high-light conditions, while nearly all 18 ABA-related genes were downregulated in NIL-33-stem (Figure 9A and Supplementary Table 8). Four key lonely guy (LOG) genes, i.e., *Glyma.01G077000, Glyma.10G053800, Glyma.12G076700*, and *Glyma.12G174900*, which are involved in CK synthesis, were significantly upregulated in NIL-33-stem; these genes result in a strong SAS phenotype under low-light conditions (Figure 9B and Supplementary Table 7). Interestingly, a few CK dehydrogenase (CKX) genes, i.e., *Glyma.04G028700, Glyma.09G063900, Glyma.13G104700*, and *Glyma.13G104600*, were significantly upregulated in the NIL-5 roots (Figure 9B and Supplementary Table 7). CKX3 and CKX5 have been proven to control the tiller strength and flag leaf senescence in monocots. These findings indicate that CKX genes may participate in the SAS process. As cell elongation activators, most GA pathway-related genes tended to be upregulated in the four tissues, but some genes were very significantly upregulated in NIL-33-stem, which is consistent with the strong SAS phenotype (Figure 9C and Supplementary Table 9). We found no significant patterns of IAA- or ethylene-related genes between the NILs under low- and high-light conditions (Supplementary Figures 5, 6 and Supplementary Tables 10, 11).

## Discussion

### Relationships between plant density tolerance, ecological adaptability and shade-avoidance

Shade avoidance and phototaxis are natural processes of green plants, but SAS can greatly reduce yields. Therefore, in production, we need crops with a moderate SAS. Plant density tolerance and ecological adaptability are two important economic traits involved in crop yields. JD17 was derived from Hobbit × Zao 5241 and has a very high yield; JD17 is the major cultivar in the Huang-Huai-Hai region of China (Qin et al., 2013). In past field production practices, JD17 showed extensive adaptability in the Huang-Huai-Hai area and was characterized as a soybean cultivar with strong lodging resistance (Zhao et al., 2015). However, the mechanism underlying these characteristics remains unclear. The two excellent traits, plant density tolerance and broad ecological adaptability, seem to be inherited in varieties by chance, but they are intrinsically related. Through years of investigation and reflection on the JD17 soybean cultivar compared to other cultivars, we inferred that both the plant density tolerance and the ecological adaptability result from the same physiological basis governing the insensitivity to light stimuli and iSAS.

Therefore, based on this inference, we think that the elite cultivar JD17 should harbor variations conferring the light-insensitive and high-density planting tolerance phenotype that had been fixed in soybean domestication and high-yield breeding.

### Putative candidate genes of shade-avoidance response 1

To determine whether there are genes or major QTLs underlying this phenotype, we confirmed the QTL mapping of SAR1 of JD17 by simulating a low-light environment using an artificial climate chamber to phenotype the RIL population. A major QTL was detected on chromosome 10 in the 1712 RIL population. Using the bulked segregant analysis sequencing (BSA-seq) method, Zeng et al. (2021) identified 408 candidate genes located on chromosomes 1, 4, 9, and 18 related to shade-tolerance traits. However, none of these genes are located on chromosome 10. Few QTL results have been published regarding shade-avoidance related phenotypes of soybean. In *Arabidopsis, ELF3* was identified as a QTL gene for the shade-avoidance response and regulated hypocotyl growth (Reed et al., 2000; Jimenez-Gomez et al., 2010; Coluccio et al., 2011). A BLASTP analysis showed that the *AtELF3* homologous genes in soybean are *Glyma.04G050200* (38.8% identity) and *Glyma.14G091900* (38.3% identity), neither of which are located on chromosome 10. Thus, the *SAR1* QTL may be driven by a previously unknown new gene. Our RNA-seq results showed that *Glyma.04G050200* was significantly downregulated [log2(FC) = –1.87, fold change = –3.66] in the NIL-5 root tissue under low-light conditions. However, there was no significant variance in the NIL-33 root tissue, and its expression could not be detected in the stem tissues of NIL-5, NIL-33, JD17, or JD12.

Notably, one of the 44 candidate genes, *Glyma10G209600.1*, was predicted to be a homolog of *AT5G04240.1* (*AtELF6*), and these two genes are highly similar, up to 73.9%. *ELF6* in *Arabidopsis* is a Jumonji N/C domain-containing protein and is supposedly a positive regulator of the expression of genes that target the BR signaling pathway and promote cell elongation (Liu et al., 2001; Yu et al., 2008; Jung et al., 2020). Based on the qRT–PCR results, *GmELF6* was significantly decreased in the stems of JD17 compared with JD12, and these results are consistent with the *ELF6* effects on cell elongation. However, the expression response levels of *Glyma10G209600.1* did not significantly differ between S5 and S33 under low-light conditions (Figure 3D).

Furthermore, ELF3 can interact with phyB to control hypocotyl elongation, plant development and flowering (Reed et al., 2000; Liu et al., 2001; Jung et al., 2020). According to our qRT–PCR results, *Glyma.15G140000.1* (*phyB*) was significantly downregulated in the stems and leaves of JD17 compared with JD12. Coincidentally, based on the qRT–PCR results, the expression of most *PIF4s* and their direct regulators (*SAURs*) was decreased in both stem and leaf tissues of JD17. *SAURs* have been hypothesized to play an important role in shade-induced cell elongation (Ren and Gray, 2015). Based on the SEM results, the hypocotyl cell elongation rate of JD17 was lower than that of JD12 in low-light (Figures 1G–J), indicating that the cell growth pathway may be affected by the *SAR1* trait. Taken together, these results indicate that the way JD17 responds to light condition changes may involve in cell elongation, which is regulated by plant hormones.

ROT4 is a key regulator involved in plant organ size; when it is overexpressed, the body axis tends to shorten (Narita et al., 2004; Ikeuchi et al., 2011). Here, we found that the *ROT4* homolog in soybean, *Glyma.10G208400*, was significantly upregulated in NIL-5 stems under low-light conditions (Figure 3D). NIL-S5 presents iSAS phenotypes under low-light conditions, which is consistent with the previous study’s conclusion. The candidate gene *Glyma.10G208400* may play an important role in the iSAS phenotype.

The dramatically upregulated NIL-S33 gene *Glyma.10G209500* encodes a threonine-protein kinase that plays roles in a multitude of cellular processes, including division, proliferation, apoptosis, and differentiation (Figure 3D). Another dramatically upregulated NIL-S33 gene, *Glyma.10G211200*, is predicted to be a type of lipase that participates in the fatty acid metabolic process. The NIL-S33 downregulated and NIL-S5 upregulated gene *Glyma.10G208300* is predicted to encode a monoglyceride acyltransferase. However, currently, there is no obvious evidence that these two genes, *Glyma.10G208300* and *Glyma.10G211200*, are related to the SAS pathway.

Taken together, *qSAR1* may be caused by a new gene that has not been reported to be related to the SAS pathway. Further fine mapping and map-based cloning of *qSAR1* could answer this interesting and important question.

### Weak shade-avoidance syndrome phenotype results from the coordinated regulation of multiple pathways throughout the genome

The SAS phenotype is regulated by multiple hormones, including auxin, CKs, GAs, BRs, ABA, strigolactones (SLs), ethylene, and the defense-related hormones SA and jasmonic acid (JA), which form a precise regulatory network (Ye et al., 2011; Yang and Li, 2017; Fernández-Milmanda and Ballaré, 2021). Photoreceptor phytochromes, cryptochromes, phototropins and pathway-related genes, such as PHYA, PHYB, PIFs, CRYs, PHOT1/2, and ARFs, are all involved in shade avoidance regulation (Franklin, 2008; Sun et al., 2013; Pierik and de Wit, 2014; Tang and Liesche, 2017; Fernández-Milmanda and Ballaré, 2021). In general, low-light conditions lead to a low allocation of resources to roots (Fernández-Milmanda and Ballaré, 2021) and significantly impact agronomic traits.

In this study, to identify the genes involved in the JD17 iSAS phenotype, a RNA-seq analysis was carried out. Interestingly, based on both the GO and KEGG enrichment analysis results, “proteasome” pathway-related genes in the iSAS-stem were significantly downregulated (Figure 6A and Supplementary Figure 4A). Proteins degraded by ubiquitin/the 26S proteasome play important roles in plant development through cellular processes and signaling pathways (Moon et al., 2004; Zhou et al., 2021). It has been proven that approximately 90% of the genes encoding components of the ubiquitin/26S proteasome pathway encode subunits of the E3 ligases in *Arabidopsis* (Moon et al., 2004; Smalle and Vierstra, 2004; Zhou et al., 2021). Constitutively Photomorphogenic 1 (COP1) is the most studied single-subunit RING E3 ubiquitin ligase and acts as a light signaling repressor that promotes SAS through the degradation of SAS-related repressors (Moon et al., 2004; Liu et al., 2021; Pierik and Ballare, 2021). In addition, two ubiquitin-specific proteases, UBP12 and UBP13, have recently been proven to promote the shade-avoidance response by enhancing PIF7 stability (Zhou et al., 2021). Consistent with the downregulation of the proteosome pathway, the negative impacts of SAS on the yield of the elite cultivar JD17 may be greatly reduced by repressing the expression of proteins that promote SAS.

Notably, in the plant hormone transduction pathway of the iSAS-root based on RNA-seq analysis, three genes were obviously upregulated. Among them, BIN2 has been proven to directly interact with phyB and regulate BR signaling and photomorphogenesis in *Arabidopsis* (Zhao et al., 2022). The hypocotyls of *BIN2 bin2-1* gain-of-function mutants were found to be shorter than those of the wild type under R and FR light. Another study indicated that BR treatment led to decreased BIN2 accumulation (Ye et al., 2011). Given the increased *BIN2* expression, we hypothesize that the iSAS of JD17 may also be affected by the BR signaling pathways that coordinate with phyB.

In this study, the qRT–PCR and transcriptome data sets showed that although most SAS-related genes were significantly regulated under low-light conditions and enriched in multiple GO terms in the stem tissues, relatively few genes were expressed specifically in the root tissue of JD17 and NIL-5 (Figure 4D), and no significantly enriched GO terms were detected in this gene set. These findings suggest that the weak SAS phenotype of JD17 may result from mechanisms including novel pathways, which is beneficial for increasing crop production. Nonetheless, the underlying mechanism is interesting, and the yield potential is worthy of in-depth exploration.

## Data availability statement

The original contributions presented in the study are publicly available. This data can be found here: NCBI, PRJNA861378.

## Author contributions

JZ, XS, LC, LY, CY, and MZ contributed to the conception and design of the study. XS, JZ, LC, QC, XT, LA, and HZ performed the experiments. XS, JZ, and LC analyzed the data. LC and JZ performed the bioinformatics analysis and revised and edited the manuscript. All authors contributed to the article and approved the submitted version.

## Footnotes

1
https://kbroman.org/qtl2/assets/vignettes/user_guide.html

2
https://phytozome-next.jgi.doe.gov/

## Supplementary material

SUPPLEMENTARY FIGURE 1 The genetic map of the1712 recombinant inbred line (RIL) population. The genetic map was constructed used the qtl package of R language.

SUPPLEMENTARY FIGURE 2 Transcriptome expression patterns of JD17, JD12, NIL-5, and NIL-33. **(A)** log2(FC) dis-tribution shown as violin plots; **(B)** venn diagram showing the intersecting relationships of the expressed gene sets in the stem tissues of NIL-5 (S5), NIL-33 (S33), JD17 (S17), and JD12 (S12) at 8 DAP; in panel (B) consensus gene expression patterns in NIL-5 stem tissue shown as volcano plots (the relationship between log2(FC) and −log10(p value)); **(C)** consensus gene expression pattern of NIL-5 root tissue shown as volcano plot [the relationship between log2(FC) and −log10(p value)].

SUPPLEMENTARY FIGURE 3 The plot plots of the GO enrichment results of special expressed gene sets: **(A)** pot plot of GO enrichment result of the special expressed genes in inactive SAS sample stem (iSAS-stem); **(B)** pot plot of GO enrichment result of the special expressed genes in strong SAS sample stem (sSAS-stem) and in strong SAS sample root (sSAS -root).

SUPPLEMENTARY FIGURE 4 KEGG enrichment results of special expressed gene sets shown by bar plots: **(A)** bar plot of KEGG enrichment result of the special expressed genes in inactive SAS sample stem (iSAS-stem) and **(B)** in inactive SAS sample root (iSAS -root); **(C)** bar plot of KEGG enrichment result of the special expressed genes in strong SAS sample stem (sSAS-stem) and (D) in strong SAS sample root (sSAS-root).

SUPPLEMENTARY FIGURE 5 The expression pattern variation of ethylene related genes in the RNA-seq transcriptome data.

SUPPLEMENTARY FIGURE 6 The expression pattern variation of IAA related genes in the RNA-seq transcriptome data.
