# Supplementary material for: Genetic and transcriptome analyses reveal the candidate genes and pathways involved in the inactive shade-avoidance response enabling high-density planting of soybean
Source: Front Plant Sci. 2022 Aug 3;13:973643. doi: 10.3389/fpls.2022.973643 (PMC9382032; doi:10.3389/fpls.2022.973643)
Supplement: Supplementary file 1 [file Data_Sheet_1.pdf]

Supplementary Tables

Table S1. The SAS related gene information used in the qRT-PCR experiment

Gene Symbol	GeneID	Forward primer (5'-3')	Reverse primer (5'-3')
**PhyB**	Glyma.15G140000.1	TCAGAATTGACAGGACTCCCAGT	GCAAGCATTCACCACTACAAAAAC
**CRY1a**	Glyma.04G101500.1	CCCTTCCCATCTATCCTTCTCAC	GTTTTGGCACTTATTACGTTTTGC
**CRY1b**	Glyma.06G103200.3	ATTCTTTGTATCTTCCCCTCCGT	CCATCAAAAATTCGTATATTGTTGC
**CRY1c**	Glyma.14G174200.2	GCTGTGGTTTGCCTATCAATTC	TTGGTGCTTTCACACACACAGAC
**CRY1d**	Glyma.13G089200.2	GCTCAGTGGTCAAAGGGAAAGG	CAACACCACAACCTCAAGAGCTAG
**CRY2a**	Glyma.10G180600.4	CGGCTAAACTTTGATAGGGTTGTG	CCAGCCTCAAACACACCTCAAAC
**CRY2b**	Glyma.02G005700.1	GTGAGACATGTGGGGAAATGC	CCTTAGATATCAGACCAACGGGTAG
**CRY2c**	Glyma.20G209900.2	ATTCTTCAGTCGCTTTTGTTTGC	CCACTTTGCTTTTATGTGAGGTATC
**PIF4a**	Glyma.02G282100.1	TCAATCCTCACAAGCACCAATAT	GCCTGACCCACAATCAACG
**PIF4c**	Glyma.18G115700.1	CTCTCCATGTGTTGATGTCACTGTC	GTTGTTGAACTAAGAAGGTTGCTAG
**PIF4d**	Glyma.14G032200.1	GCAGTGCAACATAGTCAAACAATG	GAAGAACCTGCTTTGCCACTAAC
**PIF4e**	Glyma.10G042800.4	CTTGCTATTTACAAATCCAACTGGG	CATTTGAATTTTCAGGGCTAGATTG
**PIF4f**	Glyma.11G054600.1	AGGGAGAAAGTGGTCAGTGGC	CTCATGTCACGTAGGCCAGAGG
**PIF4g**	Glyma.17G241000.1	CCCTCAGTGACAAACATAGCAAC	GTTGTATCCATAAACAGTTGTGGC
**ELF6**	Glyma.10G209600.1	GGCAAGGACAGAGCATATTCG	CTTCTCAAGCAGGAGGTTTCACA
**SAUR46**	Glyma.10G209700.1	CTCACCACTGTGCTTCTTCCAACC	GGGAAGTACGCTTTGGGAGGG
**YUC2**	Glyma.05G231100.1	CTAGCAACTGGATACAAAAGTAACG	CTTTCCAACAATGTTCAATATCTCC
**IAA16**	Glyma.07G015200.1	GCACTTCTAATTCTAAGTTGCCTTC	CTGTCATAAGCATTGATGTCCAC
**ARF16**	Glyma.10G210600.1	TCTAATGCTTCCGATGGCGTAG	GTTAGCCGCATTGATGTATTGTCTC
**NPH3**	Glyma.10G210200.1	CTCACCTTTAGACCTGGAATCAC	CTGTTTTTGGTCTAGAGATTTTTCC
**CYP2**	Glyma.12G024700.1	CGGGACCAGTGTGCTTCTTCA	CCCCTCCACTACAAAGGCTCG

Table S2. Haplotype analysis of the QTL interval region and marker detail information

Markers	SNP	Markers	SNP
**M1**	Gm10_43137790	**M8**	Gm10_44452705
**M2**	Gm10_43233286	**M9**	Gm10_44517760
**M3**	Gm10_43482211	**M10**	Gm10_44594651
**M4**	Gm10_43577420	**M11**	Gm10_44636310
**M5**	Gm10_43723377	**M12**	Gm10_44655269
**M6**	Gm10_43899046	**M13**	Gm10_44728112
**M7**	Gm10_44334211	**M14**	Gm10_45093053

Table S3. The annotated genes in the *qSAR1* internal region

GeneID	Top Uniref100 Viridaeplantae Hit
Glyma.10g207500	UniRef100_K7LKK4 VHS domain-containing protein n=2 Tax=Soja TaxID=1462606 RepID=K7LKK4_SOYBN
Glyma.10g207600	UniRef100_I1LCW4 Uncharacterized protein n=2 Tax=Soja TaxID=1462606 RepID=I1LCW4_SOYBN
Glyma.10g207700	UniRef100_I1LCX0 Glycerol-3-phosphate dehydrogenase n=1 Tax=Glycine max TaxID=3847 RepID=I1LCX0_SOYBN
Glyma.10g207800	UniRef100_I1LCX1 S4 RNA-binding domain-containing protein n=3 Tax=Soja TaxID=1462606 RepID=I1LCX1_SOYBN
Glyma.10g207900	UniRef100_A0A445IQE0 Exocyst complex component SEC8 isoform A n=2 Tax=Soja TaxID=1462606 RepID=A0A445IQE0_GLYSO
Glyma.10g208000	UniRef100_A0A0R0HWJ5 Uncharacterized protein n=1 Tax=Glycine max TaxID=3847 RepID=A0A0R0HWJ5_SOYBN
Glyma.10g208100	UniRef100_I1LCX5 Uncharacterized protein n=5 Tax=Soja TaxID=1462606 RepID=I1LCX5_SOYBN
Glyma.10g208200	UniRef100_A0A445IQC1 RING-type E3 ubiquitin transferase n=2 Tax=Soja TaxID=1462606 RepID=A0A445IQC1_GLYSO
Glyma.10g208300	UniRef100_A0A0R0I3Y8 Uncharacterized protein n=1 Tax=Glycine max TaxID=3847 RepID=A0A0R0I3Y8_SOYBN
Glyma.10g208400	UniRef100_K7LKL0 Uncharacterized protein n=2 Tax=Soja TaxID=1462606 RepID=K7LKL0_SOYBN
Glyma.10g208500	UniRef100_K7LKL1 Uncharacterized protein n=2 Tax=Soja TaxID=1462606 RepID=K7LKL1_SOYBN
Glyma.10g208600	UniRef100_I1LCY1 Uncharacterized protein n=2 Tax=Soja TaxID=1462606 RepID=I1LCY1_SOYBN
Glyma.10g208700	UniRef100_I1LCY2 S-acyltransferase n=2 Tax=Soja TaxID=1462606 RepID=I1LCY2_SOYBN
Glyma.10g208800	UniRef100_A0A445IQE3 Amino acid transporter AVT1C isoform A n=2 Tax=Soja TaxID=1462606 RepID=A0A445IQE3_GLYSO
Glyma.10g208900	UniRef100_A0A0R0HWJ6 Photosystem I reaction center subunit VIII n=1 Tax=Glycine max TaxID=3847 RepID=A0A0R0HWJ6_SOYBN
Glyma.10g209000	UniRef100_UPI00103A9A13 pumilio homolog 4-like isoform X1 n=1 Tax=Glycine soja TaxID=3848 RepID=UPI00103A9A13
Glyma.10g209100	UniRef100_I1LCY9 OTU domain-containing protein n=2 Tax=Soja TaxID=1462606 RepID=I1LCY9_SOYBN
Glyma.10g209200	UniRef100_I1LCZ0 ABC transporter domain-containing protein n=2 Tax=Soja TaxID=1462606 RepID=I1LCZ0_SOYBN
Glyma.10g209300	UniRef100_A0A445IQE1 ATPase ARSA1 isoform A n=2 Tax=Soja TaxID=1462606 RepID=A0A445IQE1_GLYSO
Glyma.10g209400	UniRef100_I1LCZ4 DNL-type domain-containing protein n=2 Tax=Soja TaxID=1462606 RepID=I1LCZ4_SOYBN
Glyma.10g209500	UniRef100_A0A445IQG4 LysM domain receptor-like kinase 3 n=1 Tax=Glycine soja TaxID=3848 RepID=A0A445IQG4_GLYSO
Glyma.10g209600	UniRef100_I1LCZ6 Uncharacterized protein n=2 Tax=Soja TaxID=1462606 RepID=I1LCZ6_SOYBN
Glyma.10g209700	UniRef100_A0A0R0I460 Uncharacterized protein n=2 Tax=Soja TaxID=1462606 RepID=A0A0R0I460_SOYBN
Glyma.10g209800	UniRef100_K7LKL7 Phenylalanine ammonia-lyase n=3 Tax=Soja TaxID=1462606 RepID=K7LKL7_SOYBN
Glyma.10g209900	UniRef100_A0A445IQH8 Vinorine synthase n=1 Tax=Glycine soja TaxID=3848 RepID=A0A445IQH8_GLYSO
Glyma.10g210000	UniRef100_I1LD00 Uncharacterized protein n=4 Tax=Soja TaxID=1462606 RepID=I1LD00_SOYBN
Glyma.10g210100	UniRef100_C6T6B6 Uncharacterized protein n=2 Tax=Soja TaxID=1462606 RepID=C6T6B6_SOYBN
Glyma.10g210200	UniRef100_A0A0R0HWP0 Uncharacterized protein n=2 Tax=Soja TaxID=1462606 RepID=A0A0R0HWP0_SOYBN
Glyma.10g210300	UniRef100_I1LD03 Dolichyl-diphosphooligosaccharide--protein glycosyltransferase subunit 1 n=2 Tax=Soja TaxID=1462606 RepID=I1LD03_SOYBN
Glyma.10g210400	UniRef100_A0A445IQF7 Protein ENHANCED DISEASE RESISTANCE 2-like isoform A n=3 Tax=Soja TaxID=1462606 RepID=A0A445IQF7_GLYSO

Glyma.10g210500	UniRef100_A0A445IQI4 GATA transcription factor n=2 Tax=Soja TaxID=1462606 RepID=A0A445IQI4_GLYSO
Glyma.10g210600	UniRef100_K7LKL9 Auxin response factor n=1 Tax=Glycine max TaxID=3847 RepID=K7LKL9_SOYBN
Glyma.10g210700	UniRef100_I1LD08 Uncharacterized protein n=3 Tax=Soja TaxID=1462606 RepID=I1LD08_SOYBN
Glyma.10g210800	UniRef100_I1LD10 N-acetyltransferase domain-containing protein n=2 Tax=Soja TaxID=1462606 RepID=I1LD10_SOYBN
Glyma.10g210900	UniRef100_K7LKM1 Peptidase_M28 domain-containing protein n=1 Tax=Glycine max TaxID=3847 RepID=K7LKM1_SOYBN
Glyma.10g211000	UniRef100_A0A0R0I4T4 Uncharacterized protein n=3 Tax=Soja TaxID=1462606 RepID=A0A0R0I4T4_SOYBN
Glyma.10g211100	UniRef100_K7LKM4 Uncharacterized protein n=2 Tax=Soja TaxID=1462606 RepID=K7LKM4_SOYBN
Glyma.10g211200	UniRef100_I1LD14 Hydrolase_4 domain-containing protein n=1 Tax=Glycine max TaxID=3847 RepID=I1LD14_SOYBN
Glyma.10g211300	UniRef100_A0A445IQW1 Patatin n=2 Tax=Soja TaxID=1462606 RepID=A0A445IQW1_GLYSO
Glyma.10g211400	UniRef100_A0A445IQI0 Chorismate synthase n=2 Tax=Soja TaxID=1462606 RepID=A0A445IQI0_GLYSO
Glyma.10g211500	UniRef100_A0A0R0HWN7 Myb_DNA-bind_3 domain-containing protein n=1 Tax=Glycine max TaxID=3847 RepID=A0A0R0HWN7_SOYBN
Glyma.10g211600	UniRef100_K7LKM5 Glutamine amidotransferase type-1 domain-containing protein n=1 Tax=Glycine max TaxID=3847 RepID=K7LKM5_SOYBN
Glyma.10g211700	UniRef100_K7LKM6 Uncharacterized protein n=2 Tax=Soja TaxID=1462606 RepID=K7LKM6_SOYBN
Glyma.10g211800	UniRef100_K7LKM8 Uncharacterized protein n=2 Tax=Soja TaxID=1462606 RepID=K7LKM8_SOYBN

Table S4. The genes with the log2FoldChange bigger than 5 in the NIL33 stem tissue (S33)

**GeneID**	**EntrezID**	**Annotation**	**log2FoldChange**
Glyma.01G03660	10052700	C2H2-type zinc finger domain-containing protein(LOC100527006)	9.858071261
Glyma.09G18880	10078963	glycine-rich cell wall structural protein(LOC100789631)	9.087210458
Glyma.01G24030	10081583	pectinesterase(LOC100815836)	8.257137369
Glyma.12G09690	10050046	cellulose synthase-like protein H1(LOC100500469)	7.326394139
Glyma.20G20230	10081912	AT-hook motif nuclear-localized protein 20(LOC100819125)	7.150628485
Glyma.02G30730	10080093	chalcone reductase CHR6(CHR6)	6.992546642
Glyma.17G03030	10079103	stress-induced protein SAM22-like(LOC100791036)	6.731884989
Glyma.15G20350	10078286	cytochrome P450 82A2(LOC100782866)	6.541597279
Glyma.13G22190	10079200	serine carboxypeptidase-like 12(LOC100792006)	6.464595583
Glyma.09G03890	10081904	transcription factor MYB13(LOC100819041)	6.378936584
Glyma.19G00830	10078861	quinone oxidoreductase-like protein At1g23740, chloroplastic-	6.278436906
Glyma.15G15610	10078941	isoflavone 2'-hydroxylase-like(LOC100789410)	6.245541621
Glyma.19G00860	10079285	2-methylene-furan-3-one reductase(LOC100792857)	6.179752008
Glyma.03G14370	10077687	3,9-dihydroxypterocarpan 6A-monooxygenase(CYP93A1)	6.095768011
Glyma.11G06260	10078060	cytochrome P450 71D8(LOC100780605)	6.073241476
Glyma.18G28580	10082016	chalcone reductase CHR5(CHR5)	5.926538704
Glyma.09G04870	10079050	isoflavone 2'-hydroxylase(LOC100790507)	5.802874609
Glyma.03G18300	10081623	auxin-responsive protein SAUR32(LOC100816234)	5.713630737
Glyma.15G22850	10052710	uncharacterized LOC100527108(LOC100527108)	5.683294995
Glyma.07G08800	10079466	glycine-rich cell wall structural protein(LOC100794667)	5.345823351
Glyma.16G16240	10078217	tryptophan aminotransferase-related protein 4(LOC100782177)	5.313528405
Glyma.15G06240	10080511	pathogenesis-related protein 1(PR1-6)	5.264780044
Glyma.19G26310	10081431	potassium transporter 5(LOC100814313)	5.253221449
Glyma.09G28190	10077949	isoliquiritigenin 2'-O-methyltransferase-like(LOC100779492)	5.211690742
Glyma.01G17790	10078197	UDP-glycosyltransferase 72E1(LOC100781979)	5.118223269
Glyma.01G22870	10078289	chalcone synthase 7(CHS7)	5.062734133

Table S5. The genes with the log2FoldChange lower than -5 in the NIL5 root tissue (R5)

GeneID	EntrezID	Annotation	log2FoldChange
Glyma.06G123200	100803495	WAT1-related protein At1g70260 (LOC100803495)	-10.11072644
Glyma.11G236500	100306576	uncharacterized LOC100306576 (LOC100306576)	-7.687150605
Glyma.08G002500	100782510	beta-galactosidase 6 (LOC100782510)	-6.873705908
Glyma.20G195100	100527435	DOG1 domain-containing protein (LOC100527435)	-6.542110982
Glyma.11G172900	NA	NA	-6.284159757
Glyma.17G080500	100811042	cytochrome P450 78A5 (LOC100811042)	-6.215177204
Glyma.11G147700	100817693	uncharacterized LOC100817693 (LOC100817693)	-5.896484679
Glyma.11G088000	100790033	3-hydroxy-3-methylglutaryl-coenzyme A reductase (LOC100790033)	-5.669497872
Glyma.20G036200	100527255	uncharacterized LOC100527255 (LOC100527255)	-5.655804587
Glyma.02G228300	100807131	mitogen-activated protein kinase kinase kinase 18(LOC100807131)	-5.357742457
Glyma.10G159700	100101867	cysteine synthase (OAS-TL6)	-5.315748141
Glyma.17G137400	100789460	21 kDa protein (LOC100789460)	-5.303776746
Glyma.13G123000	100500462	uncharacterized LOC100500462 (LOC100500462)	-5.238370588
Glyma.17G007200	100800554	cytochrome P450 71D8 (LOC100800554)	-5.226538739
Glyma.02G042700	100811784	uncharacterized LOC100811784 (LOC100811784)	-5.214947302
Glyma.14G054300	100784066	G-type lectin S-receptor-like serine/threonine-protein kinase At4g27290 (LOC100784066)	-5.042285065

Table S6. The annotation of the gene clusters with a related expression pattern to the SAS phenotype feature

Cluster	geneID	Top Uniref100 Viridaeplantae Hit
4	Glyma.03G0900	UniRef100_A0A0B2PDP5 Fimbrin-2 isoform A n=3 Tax=Soja TaxID=1462606
12	Glyma.12G0317	UniRef100_C6T2Y0 ADF-H domain-containing protein n=3 Tax=Soja TaxID=1462606
12	Glyma.11G1070	UniRef100_I1LJ16 Aa_trans domain-containing protein n=4 Tax=Soja TaxID=1462606
12	Glyma.13G0935	UniRef100_K7LWX0 Uncharacterized protein n=3 Tax=Soja TaxID=1462606
4	Glyma.11G1714	UniRef100_I1LLM2 Asparagine synthetase [glutamine-hydrolyzing] n=1 Tax=Glycine max
34	Glyma.13G0916	UniRef100_A0A0B2RR67 Uncharacterized protein n=2 Tax=Soja TaxID=1462606
4	Glyma.03G1672	UniRef100_I1JP77 TPR transcription factor n=1 Tax=Glycine max TaxID=3847
4	Glyma.08G2393	UniRef100_I1KW96 Uncharacterized protein n=1 Tax=Glycine max TaxID=3847
4	Glyma.17G0779	UniRef100_C6TNI5 X8 domain-containing protein n=2 Tax=Soja TaxID=1462606
12	Glyma.05G0105	UniRef100_C6SYK4 Uncharacterized protein n=2 Tax=Soja TaxID=1462606
12	Glyma.06G1037	UniRef100_I1K9Z2 Uncharacterized protein n=1 Tax=Glycine max TaxID=3847
12	Glyma.06G2897	UniRef100_I1KEZ0 Uncharacterized protein n=3 Tax=Soja TaxID=1462606
12	Glyma.11G0297	UniRef100_A0A445HWA6 Uncharacterized protein n=1 Tax=Glycine soja TaxID=3848
12	Glyma.11G0825	UniRef100_A0A445HYM3 Protein GDAP2-like n=2 Tax=Soja TaxID=1462606
12	Glyma.18G2539	UniRef100_A0A0B2QGW7 Protein AATF n=3 Tax=Soja TaxID=1462606
12	Glyma.19G0962	UniRef100_A0A0B2Q6E7 Uncharacterized protein n=2 Tax=Soja TaxID=1462606
12	Glyma.20G2228	UniRef100_I1NIM8 Uncharacterized protein n=2 Tax=Soja TaxID=1462606
2	Glyma.02G2783	UniRef100_I1JIV7 F-box domain-containing protein n=2 Tax=Soja TaxID=1462606
2	Glyma.06G0786	UniRef100_A0A0B2QEP2 AIG2-like protein D n=2 Tax=Soja TaxID=1462606
2	Glyma.20G0667	UniRef100_I1NEA9 Uncharacterized protein n=1 Tax=Glycine max TaxID=3847
2	Glyma.20G2111	UniRef100_I1NIB2 Uncharacterized protein n=1 Tax=Glycine max TaxID=3847
34	Glyma.11G1320	UniRef100_A0A445I148 Zinc finger A20 and AN1 domain-containing stress-associated protein 8
34	Glyma.14G1999	UniRef100_I1MBH0 Uncharacterized protein n=2 Tax=Soja TaxID=1462606
34	Glyma.18G0120	UniRef100_A0A445FMT2 Ultraviolet-B receptor UVR8 isoform A n=2 Tax=Soja TaxID=1462606
34	Glyma.18G0469	UniRef100_I1MZK7 Uncharacterized protein n=1 Tax=Glycine max TaxID=3847
34	Glyma.18G2143	UniRef100_I1N391 Dirigent protein n=1 Tax=Glycine max TaxID=3847 RepID=I1N391_SOYBN
12	Glyma.16G0911	UniRef100_A0A0B2Q644 Protein STRICTOSIDINE SYNTHASE-LIKE 10 n=2 Tax=Soja
34	Glyma.19G1040	UniRef100_A0A0R4J610 Uncharacterized protein n=1 Tax=Glycine max TaxID=3847
2	Glyma.12G0539	UniRef100_I1LQF9 Uncharacterized protein n=2 Tax=Glycine max TaxID=3847
2	Glyma.17G1006	UniRef100_C6TGD9 Uncharacterized protein n=2 Tax=Glycine max TaxID=3847
12	Glyma.07G0825	UniRef100_I1KIL5 Abhydrolase_3 domain-containing protein n=2 Tax=Soja TaxID=1462606
12	Glyma.08G0440	UniRef100_K7L4Y5 Pectinesterase n=2 Tax=Soja TaxID=1462606 RepID=K7L4Y5_SOYBN
12	Glyma.10G2451	UniRef100_A0A445ISK3 Leucine-rich repeat extensin-like protein 6 n=2 Tax=Soja TaxID=1462606
2	Glyma.04G0570	UniRef100_I1JU26 Uncharacterized protein n=2 Tax=Soja TaxID=1462606
4	Glyma.08G0771	UniRef100_I1KR76 DUF676 domain-containing protein n=2 Tax=Glycine max TaxID=3847
12	Glyma.01G2318	UniRef100_I1JAL0 Uncharacterized protein n=1 Tax=Glycine max TaxID=3847
4	Glyma.10G1727	UniRef100_A0A0R0I5R9 Uncharacterized protein n=2 Tax=Soja TaxID=1462606
12	Glyma.02G1203	UniRef100_I1JEH3 PfkB domain-containing protein n=2 Tax=Soja TaxID=1462606
12	Glyma.14G2015	UniRef100_I1MBI6 Uncharacterized protein n=1 Tax=Glycine max TaxID=3847
2	Glyma.06G0715	UniRef100_C6SYX1 Histone H2A n=2 Tax=Soja TaxID=1462606 RepID=C6SYX1_SOYBN
4	Glyma.17G0766	UniRef100_C6TJG5 t-SNARE coiled-coil homology domain-containing protein n=2 Tax=Soja
34	Glyma.15G0607	UniRef100_A0A445GQ41 Fatty acid amide hydrolase isoform A n=2 Tax=Soja TaxID=1462606
4	Glyma.20G0179	UniRef100_Q9SMF5 Major latex protein homolog n=9 Tax=Soja TaxID=1462606
34	Glyma.12G2365	UniRef100_K7LWL6 NB-ARC domain-containing protein n=2 Tax=Glycine max TaxID=3847
2	Glyma.13G3478	UniRef100_UPI0009898067 lipoxygenase n=1 Tax=Glycine max TaxID=3847
4	Glyma.08G2020	UniRef100_A0A0R0IV40 Uncharacterized protein n=2 Tax=Glycine max TaxID=3847
4	Glyma.03G1902	UniRef100_A0A445LDX5 Glucomannan 4-beta-mannosyltransferase 9 isoform A n=2 Tax=Soja
4	Glyma.19G1906	UniRef100_I1NAI2 Uncharacterized protein n=3 Tax=Soja TaxID=1462606
2	Glyma.13G1056	UniRef100_C6TJB3 Uncharacterized protein n=2 Tax=Soja TaxID=1462606
2	Glyma.13G1839	UniRef100_A0A0R0GZU6 Uncharacterized protein n=1 Tax=Glycine max TaxID=3847
2	Glyma.08G1021	UniRef100_I1KRZ2 3-oxoacyl-[acyl-carrier-protein] reductase n=2 Tax=Glycine max TaxID=3847
12	Glyma.10G0108	UniRef100_I1L7M8 AMP-binding domain-containing protein n=1 Tax=Glycine max TaxID=3847
2	Glyma.14G0587	UniRef100_I1M7S9 Fe2OG dioxygenase domain-containing protein n=2 Tax=Soja TaxID=1462606
12	Glyma.19G2613	UniRef100_I1NCS4 TF-B3 domain-containing protein n=2 Tax=Soja TaxID=1462606
34	Glyma.15G0209	UniRef100_I1MCW0 ATP-dependent 6-phosphofructokinase n=3 Tax=Soja TaxID=1462606
34	Glyma.15G2704	UniRef100_I1MJK8 Uncharacterized protein n=1 Tax=Glycine max TaxID=3847
12	Glyma.09G0303	UniRef100_I1L0L0 NAD(P)-bd_dom domain-containing protein n=2 Tax=Glycine max

4	Glyma.20G0196	UniRef100_I1NDC6 Glycine cleavage system H protein n=2 Tax=Soja TaxID=1462606
4	Glyma.19G0284	UniRef100_I1N6A5 Alpha-1,4 glucan phosphorylase n=1 Tax=Glycine max TaxID=3847
2	Glyma.06G1056	UniRef100_A0A445K7P3 2,3-bisphosphoglycerate-dependent phosphoglycerate mutase isoform A
4	Glyma.17G1281	UniRef100_I1MUM8 Malate synthase n=1 Tax=Glycine max TaxID=3847
4	Glyma.10G1376	UniRef100_A0A445IM24 ABC transporter B family member 4 isoform A n=3 Tax=Soja
12	Glyma.08G1684	UniRef100_C6T619 Uncharacterized protein n=2 Tax=Soja TaxID=1462606
2	Glyma.05G1806	UniRef100_A0A445KQ26 Inositol-3-phosphate synthase isoform A n=2 Tax=Soja TaxID=1462606
2	Glyma.16G1682	UniRef100_I1MP78 Uncharacterized protein n=1 Tax=Glycine max TaxID=3847
2	Glyma.02G2731	UniRef100_I1JIP4 3-hydroxy-3-methylglutaryl coenzyme A reductase n=3 Tax=Soja
12	Glyma.13G2639	UniRef100_A0A0R0H2B2 DIOX_N domain-containing protein n=1 Tax=Glycine max TaxID=3847
2	Glyma.04G0123	UniRef100_Q9SDZ0 Isoflavone reductase homolog 2 n=4 Tax=Soja TaxID=1462606
34	Glyma.10G2624	UniRef100_A0A0R4J4D2 PKS_ER domain-containing protein n=1 Tax=Glycine max TaxID=3847
34	Glyma.08G1317	UniRef100_I1KSX3 Lipase_GDSL domain-containing protein n=2 Tax=Soja TaxID=1462606
12	Glyma.20G2480	UniRef100_A0A0B2R4K6 Sn1-specific diacylglycerol lipase alpha n=3 Tax=Soja TaxID=1462606
2	Glyma.10G2003	UniRef100_I1LCP5 WD_REPEATS_REGION domain-containing protein n=1 Tax=Glycine max
2	Glyma.02G1371	UniRef100_I1JEZ1 Sas10 domain-containing protein n=2 Tax=Glycine max TaxID=3847
2	Glyma.09G0946	UniRef100_I1L2A8 Uncharacterized protein n=4 Tax=Soja TaxID=1462606
34	Glyma.01G0631	UniRef100_A0A0B2PDN6 Salicylate O-methyltransferase n=2 Tax=Soja TaxID=1462606
34	Glyma.06G1548	UniRef100_I1KBK8 Uncharacterized protein n=2 Tax=Soja TaxID=1462606
12	Glyma.17G2448	UniRef100_I1MXR5 Uncharacterized protein n=2 Tax=Soja TaxID=1462606
2	Glyma.16G2103	UniRef100_K7MIT4 Uncharacterized protein n=2 Tax=Soja TaxID=1462606
34	Glyma.02G0415	UniRef100_A0A445LJC8 MADS-box protein SVP isoform A n=4 Tax=Soja TaxID=1462606
4	Glyma.08G2001	UniRef100_P10743 Stem 31 kDa glycoprotein n=4 Tax=Soja TaxID=1462606
4	Glyma.13G2003	UniRef100_I1M0V4 Uncharacterized protein n=2 Tax=Soja TaxID=1462606
4	Glyma.15G2392	UniRef100_C6T741 Uncharacterized protein n=1 Tax=Glycine max TaxID=3847
4	Glyma.17G0639	UniRef100_A0A445G2U0 AAA-ATPase n=2 Tax=Soja TaxID=1462606
4	Glyma.17G1402	UniRef100_C6T126 AAI domain-containing protein n=2 Tax=Soja TaxID=1462606
12	Glyma.02G1805	UniRef100_A0A0B2PJS2 Uncharacterized protein n=2 Tax=Soja TaxID=1462606
12	Glyma.02G2924	UniRef100_K7KBG7 Uncharacterized protein n=2 Tax=Soja TaxID=1462606
12	Glyma.06G1326	UniRef100_I1KAW9 Rhodanese domain-containing protein n=3 Tax=Soja TaxID=1462606
12	Glyma.07G0872	UniRef100_I1KIS0 Uncharacterized protein n=2 Tax=Soja TaxID=1462606
12	Glyma.07G2087	NA
12	Glyma.15G0498	UniRef100_I1MDT0 WAT1-related protein n=2 Tax=Soja TaxID=1462606
2	Glyma.02G0326	UniRef100_K7K671 WAT1-related protein n=1 Tax=Glycine max TaxID=3847
2	Glyma.04G0572	UniRef100_I1K8I2 Smr domain-containing protein n=2 Tax=Soja TaxID=1462606
2	Glyma.09G0063	UniRef100_K7LB55 Ala_racemase_N domain-containing protein n=1 Tax=Glycine max
2	Glyma.09G0176	UniRef100_I1L079 Uncharacterized protein n=2 Tax=Soja TaxID=1462606
2	Glyma.14G0696	UniRef100_K7M5C9 Arm_2 domain-containing protein n=2 Tax=Soja TaxID=1462606
2	Glyma.15G0025	UniRef100_A0A0B2QLW6 Uncharacterized protein n=2 Tax=Soja TaxID=1462606
2	Glyma.15G1268	UniRef100_I1MFZ9 Uncharacterized protein n=2 Tax=Soja TaxID=1462606
34	Glyma.07G1899	UniRef100_I1KLC9 EF-hand domain-containing protein n=2 Tax=Soja TaxID=1462606
34	Glyma.08G2111	UniRef100_A0A0B2SU86 VOC domain-containing protein n=2 Tax=Soja TaxID=1462606
34	Glyma.13G0906	UniRef100_A0A0B2RRA0 Uncharacterized protein n=2 Tax=Soja TaxID=1462606
34	Glyma.16G0209	UniRef100_A0A445GD54 Uncharacterized protein n=2 Tax=Soja TaxID=1462606
34	Glyma.18G2826	UniRef100_K7MV88 Dirigent protein n=1 Tax=Glycine max TaxID=3847
34	Glyma.20G0263	NA
2	Glyma.12G1769	UniRef100_I1LTL8 MFS domain-containing protein n=2 Tax=Soja TaxID=1462606
2	Glyma.07G1157	UniRef100_A0A445JVI3 Protein mago nashi-like 2 isoform A n=2 Tax=Soja TaxID=1462606
34	Glyma.09G1176	UniRef100_A0A0B2QEE2 Nudix hydrolase 25 n=2 Tax=Soja TaxID=1462606
4	Glyma.11G1902	UniRef100_I1LKW7 Epimerase domain-containing protein n=2 Tax=Soja TaxID=1462606
2	Glyma.04G1863	UniRef100_I1JXA2 Uncharacterized protein n=3 Tax=Glycine max TaxID=3847
2	Glyma.09G2791	UniRef100_I1L750 Uncharacterized protein n=2 Tax=Glycine max TaxID=3847
2	Glyma.12G0926	UniRef100_A0A0R0HCQ2 Uncharacterized protein n=2 Tax=Soja TaxID=1462606
34	Glyma.13G2617	UniRef100_A0A0R0H2Z4 Uncharacterized protein n=1 Tax=Glycine max TaxID=3847
12	Glyma.09G1895	UniRef100_A0A0B2SLB7 Aldehyde dehydrogenase family 2 member C4 n=2 Tax=Soja
4	Glyma.07G1027	UniRef100_A0A0B2SK06 Phosphoethanolamine N-methyltransferase n=2 Tax=Soja
12	Glyma.02G1456	UniRef100_UPI000E21C02F type I inositol polyphosphate 5-phosphatase 4 isoform X1 n=1

12	Glyma.11G0510	UniRef100_I1LH91 AB hydrolase-1 domain-containing protein n=2 Tax=Soja TaxID=1462606
12	Glyma.17G0722	UniRef100_A0A0R0FI80 Cellulose synthase n=1 Tax=Glycine max TaxID=3847
12	Glyma.05G1619	UniRef100_C6TL69 FAS1 domain-containing protein n=1 Tax=Glycine max TaxID=3847
12	Glyma.02G1054	UniRef100_I1JE25 Polysacc_synt_4 domain-containing protein n=2 Tax=Soja TaxID=1462606
12	Glyma.05G0078	UniRef100_A0A445KHJ8 Protein KINESIN LIGHT CHAIN-RELATED 2 isoform A n=2
2	Glyma.06G0509	UniRef100_A0A0B2RG16 PPM-type phosphatase domain-containing protein n=2 Tax=Soja
2	Glyma.10G2829	UniRef100_I1LF55 PPM-type phosphatase domain-containing protein n=2 Tax=Soja
2	Glyma.20G1068	UniRef100_A0A445F389 PPM-type phosphatase domain-containing protein n=3 Tax=Soja
34	Glyma.04G0590	UniRef100_A0A0R4J346 PPM-type phosphatase domain-containing protein n=2 Tax=Soja
34	Glyma.09G2007	UniRef100_I1L4T1 Exostosin domain-containing protein n=2 Tax=Soja TaxID=1462606
2	Glyma.08G1149	UniRef100_I1KSC8 Uncharacterized protein n=1 Tax=Glycine max TaxID=3847
2	Glyma.02G1507	UniRef100_I1JFE1 Protein kinase domain-containing protein n=1 Tax=Glycine max TaxID=3847
12	Glyma.07G0702	UniRef100_I1KI96 Protein kinase domain-containing protein n=1 Tax=Glycine max TaxID=3847
2	Glyma.05G0320	UniRef100_A0A445KIN3 Serine/threonine-protein kinase AtPK2/AtPK19 isoform C n=2 Tax=Soja
2	Glyma.10G0645	UniRef100_I1L958 Uncharacterized protein n=2 Tax=Glycine max TaxID=3847
2	Glyma.12G0404	UniRef100_A0A0R0HAI4 Uncharacterized protein n=3 Tax=Glycine max TaxID=3847
2	Glyma.13G3578	UniRef100_I1M5K1 Uncharacterized protein n=2 Tax=Soja TaxID=1462606
34	Glyma.06G0848	UniRef100_I1K9D1 RING-type domain-containing protein n=2 Tax=Soja TaxID=1462606
34	Glyma.13G1159	UniRef100_I1LYC2 RING-type domain-containing protein n=1 Tax=Glycine max TaxID=3847
4	Glyma.17G0165	UniRef100_I1MR85 Peptidase A1 domain-containing protein n=2 Tax=Soja TaxID=1462606
4	Glyma.08G2910	UniRef100_I1KXK2 Cyclin N-terminal domain-containing protein n=3 Tax=Soja TaxID=1462606
34	Glyma.19G0192	UniRef100_K7MW11 Plasma membrane ATPase n=1 Tax=Glycine max TaxID=3847
4	Glyma.08G1488	UniRef100_K7L6T0 Homeobox domain-containing protein n=2 Tax=Soja TaxID=1462606
12	Glyma.13G3685	UniRef100_I1M5X6 BHLH domain-containing protein n=2 Tax=Glycine max TaxID=3847
2	Glyma.01G0491	UniRef100_K7K1V4 HTH myb-type domain-containing protein n=3 Tax=Soja TaxID=1462606
2	Glyma.17G2168	UniRef100_A0A0B2QM64 Zinc finger protein 3 n=2 Tax=Soja TaxID=1462606
34	Glyma.15G1520	UniRef100_I1MGP4 AP2-EREBP transcription factor n=1 Tax=Glycine max TaxID=3847
12	Glyma.20G0604	UniRef100_K7N1D6 CONSTANS-like zinc finger protein n=1 Tax=Glycine max TaxID=3847
34	Glyma.07G0481	UniRef100_I1KHM3 NAC domain-containing protein n=1 Tax=Glycine max TaxID=3847
2	Glyma.05G1799	UniRef100_I1K4K5 AP2/ERF domain-containing protein n=1 Tax=Glycine max TaxID=3847
4	Glyma.16G0476	UniRef100_I1ML94 AP2/ERF domain-containing protein n=1 Tax=Glycine max TaxID=3847
12	Glyma.12G2339	UniRef100_K7LWJ8 Uncharacterized protein n=3 Tax=Soja TaxID=1462606
4	Glyma.17G0752	UniRef100_UPI0003DED04C transcription factor bHLH69 isoform X1 n=1 Tax=Glycine max
34	Glyma.15G2039	UniRef100_I1MIE1 BHLH domain-containing protein n=1 Tax=Glycine max TaxID=3847
34	Glyma.17G0752	UniRef100_UPI0003DED04C transcription factor bHLH69 isoform X1 n=1 Tax=Glycine max
34	Glyma.06G0596	UniRef100_A0A0R4J3G9 CONSTANS-like zinc finger protein n=3 Tax=Soja TaxID=1462606
12	Glyma.06G0291	UniRef100_A0A445K3X1 BEL1-like homeodomain protein 3 isoform A n=2 Tax=Soja
34	Glyma.16G0264	UniRef100_I1MKL2 WRKY transcription factor n=2 Tax=Soja TaxID=1462606
2	Glyma.11G0960	UniRef100_A0A445I0A0 Scarecrow-like protein 3 isoform B n=4 Tax=Soja TaxID=1462606
12	Glyma.07G1268	UniRef100_I1KJS9 Uncharacterized protein n=2 Tax=Soja TaxID=1462606
34	Glyma.06G1140	UniRef100_I1KAA6 NAC transcription factor n=2 Tax=Soja TaxID=1462606
2	Glyma.08G2107	UniRef100_A0A0B2SU90 F-box only protein 6 n=1 Tax=Glycine soja TaxID=3848
2	Glyma.13G1143	UniRef100_I1LYA4 Eukaryotic translation initiation factor 3 subunit K n=1 Tax=Glycine max
34	Glyma.17G2582	UniRef100_A0A0R0FV47 Uncharacterized protein n=2 Tax=Soja TaxID=1462606
2	Glyma.16G1703	UniRef100_I1MPA0 RING-type E3 ubiquitin transferase n=1 Tax=Glycine max TaxID=3847
4	Glyma.01G1026	UniRef100_I1J6Z7 Aldo_ket_red domain-containing protein n=2 Tax=Soja TaxID=1462606
4	Glyma.08G1362	UniRef100_C6TFT8 Annexin n=3 Tax=Soja TaxID=1462606 RepID=C6TFT8_SOYBN
4	Glyma.09G2145	UniRef100_C6SXV7 ACB domain-containing protein n=1 Tax=Glycine max TaxID=3847
12	Glyma.08G0829	UniRef100_C6TNE6 Chlorophyll a-b binding protein, chloroplastic n=2 Tax=Soja TaxID=1462606
34	Glyma.17G2053	UniRef100_I1MWM9 Fe2OG dioxygenase domain-containing protein n=2 Tax=Soja
34	Glyma.03G1010	UniRef100_A0A445L9W7 ABC transporter C family member 9 isoform A n=2 Tax=Soja
34	Glyma.01G1711	UniRef100_I1J8R4 Peroxidase n=2 Tax=Soja TaxID=1462606 RepID=I1J8R4_SOYBN
2	Glyma.07G1408	UniRef100_A0A0B2PBV1 Universal stress protein A-like protein n=3 Tax=Soja TaxID=1462606
12	Glyma.19G2500	UniRef100_I1NCF1 Lipase_GDSL domain-containing protein n=2 Tax=Soja TaxID=1462606
4	Glyma.06G2054	UniRef100_I1KD34 COBRA-like protein n=3 Tax=Soja TaxID=1462606 RepID=I1KD34_SOYBN
34	Glyma.08G3379	UniRef100_A0A0B2P2E2 BTB/POZ domain-containing protein n=2 Tax=Soja TaxID=1462606
2	Glyma.10G2341	UniRef100_A0A0B2QQ21 Protein NLP7 n=2 Tax=Soja TaxID=1462606
34	Glyma.06G0108	UniRef100_A0A0R0JDY5 Uncharacterized protein n=1 Tax=Glycine max TaxID=3847
12	Glyma.13G1677	UniRef100_I1LZZ3 Uncharacterized protein n=2 Tax=Soja TaxID=1462606
2	Glyma.16G1651	UniRef100_A0A0B2SAL0 Notchless protein-like n=2 Tax=Soja TaxID=1462606

34	Glyma.10G1702	UniRef100_I1LBV2 Protein pelota homolog n=2 Tax=Soja TaxID=1462606
2	Glyma.02G0641	UniRef100_I1JCX4 Uncharacterized protein n=2 Tax=Soja TaxID=1462606
34	Glyma.09G2332	UniRef100_K7LFM5 Uncharacterized protein n=2 Tax=Soja TaxID=1462606
34	Glyma.05G1053	UniRef100_K7KPG2 Adenylyl-sulfate kinase n=2 Tax=Soja TaxID=1462606
34	Glyma.02G1451	UniRef100_A0A0B2QGF8 Sulfate transporter 3.1 n=2 Tax=Soja TaxID=1462606
2	Glyma.13G3718	UniRef100_A0A0B2RHS5 Uncharacterized protein n=2 Tax=Soja TaxID=1462606
12	Glyma.12G0204	UniRef100_A0A0B2QK98 ABC transporter G family member 15 n=2 Tax=Soja TaxID=1462606
2	Glyma.13G0508	UniRef100_I1LWX8 RING-type domain-containing protein n=1 Tax=Glycine max TaxID=3847
4	Glyma.17G1325	UniRef100_I1MUS3 RmlD_sub_bind domain-containing protein n=1 Tax=Glycine max
12	Glyma.12G1004	UniRef100_K7LU00 Uncharacterized protein n=2 Tax=Soja TaxID=1462606

Table S7. The information of cytokinin (CK) related genes used in this study

No.	Gene ID	Annotation
**1**	Glyma.09G063900	cytokinin dehydrogenase 3 (LOC100797928)
**2**	Glyma.04G028700	cytokinin dehydrogenase 5 (LOC100790584)
**3**	Glyma.11G149100	cytokinin dehydrogenase 6 (LOC100797430)
**4**	Glyma.09G225400	cytokinin dehydrogenase 6 (LOC100800722)
**5**	Glyma.10G078500	cytokinin riboside 5'-monophosphate phosphoribohydrolase LOG7 (LOC100810349)
**6**	Glyma.13G324700	cytokinin riboside 5'-monophosphate phosphoribohydrolase LOG1 (LOC100811156)
**7**	Glyma.20G159700	cytokinin hydroxylase (LOC100785284)
**8**	Glyma.14G218100	cytokinin riboside 5'-monophosphate phosphoribohydrolase LOG1-like (LOC100785143)
**9**	Glyma.13G104600	cytokinin dehydrogenase 3 (LOC100816815)
**10**	Glyma.19G198400	cytokinin riboside 5'-monophosphate phosphoribohydrolase LOG7 (LOC100788087)
**11**	Glyma.08G325100	probable cytokinin riboside 5'-monophosphate phosphoribohydrolase LOGL3 (LOC100803538)
**12**	Glyma.13G104700	cytokinin dehydrogenase 3 (LOC100782645)
**13**	Glyma.19G135100	cytokinin dehydrogenase 1 (LOC100777568)
**14**	Glyma.12G076700	cytokinin riboside 5'-monophosphate phosphoribohydrolase LOG1(LOC100815735)
**15**	Glyma.12G174900	cytokinin riboside 5'-monophosphate phosphoribohydrolase LOG1(LOC100780273)
**16**	Glyma.09G137600	cytokinin hydroxylase (LOC100784128)
**17**	Glyma.08G365000	cytokinin hydroxylase (LOC100812443)
**18**	Glyma.16G109500	cytokinin riboside 5'-monophosphate phosphoribohydrolase LOG3(LOC102666174)
**19**	Glyma.17G054600	cytokinin dehydrogenase 3 (LOC100808553)
**20**	Glyma.06G053000	cytokinin riboside 5'-monophosphate phosphoribohydrolase LOG8(LOC100785484)
**21**	Glyma.10G234600	cytokinin hydroxylase (LOC100802882)
**22**	Glyma.17G054500	cytokinin dehydrogenase 3-like (CKX3)
**23**	Glyma.10G234700	cytokinin hydroxylase (LOC100803409)
**24**	Glyma.01G077000	cytokinin induced message (CIM1)
**25**	Glyma.10G053800	cytokinin riboside 5'-monophosphate phosphoribohydrolase LOG1 (LOC100802378)
**26**	Glyma.09G063500	cytokinin dehydrogenase 3 (LOC100793158)
**27**	Glyma.09G229100	cytokinin riboside 5'-monophosphate phosphoribohydrolase LOG1 (LOC100811032)
**28**	Glyma.16G182600	cytokinin hydroxylase (LOC100810679)
**29**	Glyma.06G025200	cytokinin riboside 5'-monophosphate phosphoribohydrolase LOG1 (LOC100815440)
**30**	Glyma.12G011400	cytokinin dehydrogenase 6 (LOC100808619)
**31**	Glyma.15G170300	cytokinin dehydrogenase 3 (LOC100804431)

**32**	Glyma.19G182100	cytokinin riboside 5'-monophosphate phosphoribohydrolase LOG3 (LOC100810030)
**33**	Glyma.02G198300	cytokinin riboside 5'-monophosphate phosphoribohydrolase LOG7 (LOC100816928)
**34**	Glyma.03G200800	cytokinin riboside 5'-monophosphate phosphoribohydrolase LOG7 (LOC100792663)
**35**	Glyma.04G025100	probable cytokinin riboside 5'-monophosphate phosphoribohydrolase LOGL10 (LOC100782787)
**36**	Glyma.17G257200	cytokinin riboside 5'-monophosphate phosphoribohydrolase LOG1-like (LOC100814612)
**37**	Glyma.10G089000	cytokinin riboside 5'-monophosphate phosphoribohydrolase LOG1 (LOC100783827)
**38**	Glyma.03G181300	cytokinin riboside 5'-monophosphate phosphoribohydrolase LOG3-like (LOC100787375)
**39**	Glyma.18G297200	cytokinin hydroxylase (LOC100808392)
**40**	Glyma.13G046400	cytokinin hydroxylase (LOC100812567)
**41**	Glyma.17G227200	cytokinin riboside 5'-monophosphate phosphoribohydrolase LOG8 (LOC100819765)
**42**	Glyma.03G133300	cytokinin dehydrogenase 1-like (CKX1)
**43**	Glyma.14G099000	cytokinin dehydrogenase 7 (LOC100783374)
**44**	Glyma.11G145100	cytokinin riboside 5'-monophosphate phosphoribohydrolase LOG1 (LOC100819644)
**45**	Glyma.12G002400	cytokinin-regulated kinase (LOC100305418)
**46**	Glyma.13G140900	cytokinin riboside 5'-monophosphate phosphoribohydrolase LOG3-like (LOC100791145)

Table S8. The information of abscisic acid (ABA) related genes used in this study

No.	Gene ID	Annotation
**1**	Glyma.09G176900	protein C2-DOMAIN ABA-RELATED 4(LOC100810504)
**2**	Glyma.05G246400	protein C2-DOMAIN ABA-RELATED 4(LOC100775652)
**3**	Glyma.18G202900	protein RESPONSE TO ABA AND SALT 1(LOC100794752)
**4**	Glyma.09G175600	protein C2-DOMAIN ABA-RELATED 4(LOC100781273)
**5**	Glyma.07G268400	protein C2-DOMAIN ABA-RELATED 11(LOC100798217)
**6**	Glyma.11G216900	protein C2-DOMAIN ABA-RELATED 4-like(LOC100306214)
**7**	Glyma.07G219900	protein C2-DOMAIN ABA-RELATED 7(LOC100781585)
**8**	Glyma.18G039700	protein C2-DOMAIN ABA-RELATED 1(LOC100793531)
**9**	Glyma.08G365700	protein ABA DEFICIENT 4, chloroplastic(LOC100814584)
**10**	Glyma.04G124200	ABA-RESPONSIVE ELEMENT BINDING PROTEIN3(AREB3-1)
**11**	Glyma.06G314400	ABA-RESPONSIVE ELEMENT BINDING PROTEIN3(AREB3-2)
**12**	Glyma.07G151600	protein RESPONSE TO ABA AND SALT 1(LOC100780874)
**13**	Glyma.07G102400	protein C2-DOMAIN ABA-RELATED 4(LOC100779437)
**14**	Glyma.01G063100	salicylic acid methyl transferase-like protein (SABATH1)
**15**	Glyma.12G119100	protein C2-DOMAIN ABA-RELATED 11(LOC100802784)
**16**	Glyma.08G054500	protein C2-DOMAIN ABA-RELATED 4(LOC100799469)
**17**	Glyma.19G070800	protein C2-DOMAIN ABA-RELATED 7(LOC100784381)
**18**	Glyma.18G296600	protein ABA DEFICIENT 4, chloroplastic (LOC100806791)

Table S9. The information of gibberellin (GA) related genes used in this study

No.	Gene ID	Annotation
**1**	Glyma.11G003200	gibberellin 2-beta-dioxygenase 4(GA2OX4)
**2**	Glyma.15G093900	gibberellin 2-beta-dioxygenase 8(GA2OX8)
**3**	Glyma.17G258100	gibberellin-regulated protein 28(GASA28)
**4**	Glyma.17G258200	gibberellin-regulated protein 29(GASA29)
**5**	Glyma.18G061300	gibberellin 2-beta-dioxygenase 8(LOC100819244)
**6**	Glyma.10G010700	gibberellin 2-beta-dioxygenase 2(GA2OX2)
**7**	Glyma.07G236100	gibberellin 2-beta-dioxygenase 2(LOC100783917)
**8**	Glyma.04G244200	gibberellin 20 oxidase 2(LOC100776707)
**9**	Glyma.12G214000	gibberellin 2-beta-dioxygenase 8(LOC100790757)
**10**	Glyma.13G069900	gibberellin-regulated protein 21(GASA21)
**11**	Glyma.17G092800	gibberellin-regulated protein 26(GASA26)
**12**	Glyma.19G022500	gibberellin-regulated protein 34(GASA34)
**13**	Glyma.10G123000	gibberellin 2-beta-dioxygenase 3(GA2OX3)
**14**	Glyma.13G035600	gibberellin 20 oxidase 5(GA20OX5)
**15**	Glyma.04G024400	gibberellin-regulated protein 4(GASA4)
**16**	Glyma.10G259800	gibberellin-regulated protein 17(GASA17)
**17**	Glyma.19G218700	gibberellin 2-beta-dioxygenase 2(LOC100777233)
**18**	Glyma.06G193800	gibberellin-regulated protein 11(GASA11)
**19**	Glyma.17G145200	gibberellin 2-beta-dioxygenase 8(LOC106796737)
**20**	Glyma.09G259600	gibberellin 3-beta-dioxygenase 4-like(LOC100812108)
**21**	Glyma.17G037300	gibberellin 2-beta-dioxygenase 2(LOC100816206)
**22**	Glyma.19G013000	gibberellin-regulated protein 33(GASA33)
**23**	Glyma.10G216000	gibberellin-regulated protein 15(GASA15)
**24**	Glyma.08G085400	gibberellin 2-beta-dioxygenase(LOC100776094)
**25**	Glyma.08G291900	gibberellin-regulated protein 12(GASA12)
**26**	Glyma.13G285400	chitin-inducible gibberellin-responsive protein 1(LOC100799073)
**27**	Glyma.20G141200	gibberellin 2-beta-dioxygenase 8(LOC100792172)
**28**	Glyma.06G119100	gibberellin 20 oxidase 2-like(LOC106794148)
**29**	Glyma.10G216100	gibberellin-regulated protein 16(GASA16)
**30**	Glyma.02G245600	gibberellin-regulated protein 1(GASA1)
**31**	Glyma.04G211100	gibberellin 20 oxidase 2-like(LOC100777070)
**32**	Glyma.03G148300	gibberellin receptor GID1B(LOC100788435)
**33**	Glyma.20G131100	gibberellin-regulated protein 36(GASA36)
**34**	Glyma.08G208300	gibberellin 3-beta-dioxygenase 1(LOC100794180)
**35**	Glyma.11G104800	gibberellin 2-beta-dioxygenase 6(LOC100819653)
**36**	Glyma.02G010100	gibberellin 2-beta-dioxygenase 1(GA2OX1)
**37**	Glyma.04G169600	gibberellin-regulated protein 5(GASA5)
**38**	Glyma.20G175800	gibberellin-regulated protein 37(GASA37)

**39**	Glyma.09G238300	gibberellin-regulated protein 14(GASA14)
**40**	Glyma.06G185300	gibberellin-regulated protein 10(GASA10)
**41**	Glyma.18G259400	gibberellin-regulated protein 32(GASA32)
**42**	Glyma.20G230600	gibberellin receptor GID1C(GID1C2)
**43**	Glyma.02G151100	gibberellin receptor GID1B(LOC100804463)
**44**	Glyma.04G179500	gibberellin-regulated protein 6(GASA6)
**45**	Glyma.14G219100	gibberellin-regulated protein 24(GASA24)
**46**	Glyma.02G136000	gibberellin 20 oxidase 1-like(LOC100807298)
**47**	Glyma.02G136200	gibberellin 20 oxidase 1-like(LOC100809985)
**48**	Glyma.13G039600	gibberellin-regulated protein 18(GASA18)
**49**	Glyma.13G259400	gibberellin 2-beta-dioxygenase 6(GA2OX6)
**50**	Glyma.11G172500	gibberellin 2-beta-dioxygenase 8-like(LOC100794065)
**51**	Glyma.13G259500	gibberellin 2-beta-dioxygenase 7(GA2OX7)
**52**	Glyma.13G039300	gibberellin-regulated protein 18(GASA18)
**53**	Glyma.17G205300	gibberellin 3-beta-dioxygenase 6(GA3OX6)
**54**	Glyma.06G072600	gibberellin 3-beta-dioxygenase 2(GA3OX2)
**55**	Glyma.18G132100	gibberellin-regulated protein 31(GASA31)
**56**	Glyma.03G131700	gibberellin-regulated protein 3(GASA3)
**57**	Glyma.05G034500	gibberellin-regulated protein 7(GASA7)
**58**	Glyma.10G022900	gibberellin receptor GID1B(LOC100812133)
**59**	Glyma.14G215600	gibberellin-regulated protein 23(GASA23)
**60**	Glyma.14G087200	gibberellin-regulated protein 22(GASA22)
**61**	Glyma.04G071000	gibberellin 3-beta-dioxygenase 1(GA3OX1)
**62**	Glyma.07G033800	gibberellin 3-beta-dioxygenase 1(LOC100795921)
**63**	Glyma.09G032200	gibberellin 2-beta-dioxygenase 2(LOC100811043)
**64**	Glyma.13G288000	gibberellin 2-beta-dioxygenase 8(LOC100786176)
**65**	Glyma.09G095200	gibberellin-regulated protein 13(GASA13)
**66**	Glyma.13G361700	gibberellin 3-beta-dioxygenase 3(GA3OX3)
**67**	Glyma.13G048500	gibberellin 3-beta-dioxygenase 1-like(LOC100783522)
**68**	Glyma.09G149200	gibberellin 20-oxidase(GA20OX3)
**69**	Glyma.12G029800	gibberellin 2-beta-dioxygenase 6(LOC100809329)
**70**	Glyma.06G044400	gibberellin-regulated protein 9(GASA9)
**71**	Glyma.13G287600	gibberellin 2-beta-dioxygenase 8(LOC100805270)
**72**	Glyma.06G024500	gibberellin-regulated protein 8(GASA8)
**73**	Glyma.03G009600	gibberellin 20-oxidase-like protein(LOC100782766)
**74**	Glyma.13G218200	gibberellin 2-beta-dioxygenase 5(GA2OX5)
**75**	Glyma.03G019800	gibberellin 20 oxidase 1(GA20OX1)
**76**	Glyma.20G153400	gibberellin 20 oxidase 8(GA20OX8)
**77**	Glyma.17G178300	gibberellin 2-beta-dioxygenase 8(LOC106796618)
**78**	Glyma.19G133600	gibberellin-regulated protein 35(GASA35)
**79**	Glyma.12G213700	gibberellin 2-beta-dioxygenase 8(LOC106795437)
**80**	Glyma.15G012100	gibberellin 3-beta-dioxygenase 5(GA3OX5)

**81**	Glyma.14G157400	gibberellin 20 oxidase 6(GA20OX6)
**82**	Glyma.07G081700	gibberellin 20 oxidase 2(GA20OX2)
**83**	Glyma.10G158000	gibberellin receptor GID1C(LOC100815161)
**84**	Glyma.16G200800	gibberellin 20-oxidase 7(GA20OX7)
**85**	Glyma.17G237100	gibberellin-regulated protein 27(GASA27)
**86**	Glyma.15G252100	gibberellin 2-beta-dioxygenase 10(GA2OX10)
**87**	Glyma.14G128400	gibberellin 3-beta-dioxygenase 4(GA3OX4)
**88**	Glyma.05G081600	gibberellin 2-beta-dioxygenase 8(LOC100793950)
**89**	Glyma.03G221600	gibberellin 2-beta-dioxygenase 2(LOC100796690)
**90**	Glyma.05G130600	gibberellin 2-beta-dioxygenase(LOC102661568)

Table S10. The information of Indole-3-acetic acid (IAA) related genes used in this study

No.	Gene ID	Annotation
1	Glyma.08G197100	IAA-amino acid hydrolase ILR1-like(LOC100790664)
2	Glyma.08G197200	IAA-amino acid hydrolase ILR1-like 1(LOC100818503)
3	Glyma.08G197300	IAA-amino acid hydrolase ILR1-like 4(LOC100819037)
4	Glyma.08G197000	IAA-amino acid hydrolase ILR1-like 4(LOC100790140)
5	Glyma.19G168500	auxin-responsive protein IAA13-like(LOC100813788)
6	Glyma.17G112300	auxin-responsive protein IAA13-like(LOC100804096)
7	Glyma.10G162400	auxin-responsive protein IAA16-like(LOC100781847)
8	Glyma.03G167400	auxin-responsive protein IAA10-like(LOC100789143)
9	Glyma.07G015200	auxin-responsive protein IAA16(LOC100793822)
10	Glyma.10G270500	auxin-responsive protein IAA32(LOC100777024)
11	Glyma.10G040400	auxin-responsive protein IAA11(LOC100527033)
12	Glyma.08G203100	auxin-responsive protein IAA27-like(LOC100804782)
13	Glyma.06G115100	IAA-amino acid hydrolase ILR1-like 6(LOC100785666)
14	Glyma.08G036400	auxin-responsive protein IAA27-like(LOC100795749)
15	Glyma.13G354100	auxin-responsive protein IAA26(LOC100799244)
16	Glyma.03G247400	auxin-responsive protein IAA16(LOC100796888)
17	Glyma.04G047500	uncharacterized protein KIAA0930 homolog(LOC100796388)
18	Glyma.06G067700	auxin-responsive protein IAA28(LOC100783359)
19	Glyma.20G210500	AUX/IAA family protein(LOC100527777)
20	Glyma.20G210400	auxin-responsive protein IAA14(LOC100810762)
21	Glyma.13G117100	auxin-responsive protein IAA4(LOC100818083)
22	Glyma.14G185400	auxin-responsive protein IAA9(LOC100815447)
23	Glyma.05G137400	IAA-alanine resistance protein 1(LOC100816483)
24	Glyma.13G127000	auxin-responsive protein IAA11(LOC100779400)
25	Glyma.20G225000	AUX/IAA family protein(LOC100499872)
26	Glyma.08G292000	IAA-amino acid hydrolase ILR1-like 4(LOC100780711)
27	Glyma.15G020300	auxin-responsive protein IAA26(LOC100804593)
28	Glyma.09G203300	auxin-responsive protein IAA8(LOC100785203)
29	Glyma.04G089900	auxin-responsive protein IAA8-like(LOC100783314)
30	Glyma.01G019400	AUX/IAA family protein(GH1)
31	Glyma.18G132000	IAA-amino acid hydrolase ILR1-like 4(LOC100788267)
32	Glyma.09G193000	auxin-responsive protein IAA27(LOC100803754)
33	Glyma.15G017500	auxin-responsive protein IAA27-like(LOC100804423)
34	Glyma.13G352400	IAA-amino acid hydrolase ILR1-like 4(LOC100794121)
35	Glyma.08G092700	IAA-alanine resistance protein 1(LOC100786061)
36	Glyma.07G018100	auxin-responsive protein IAA27(LOC100795203)
37	Glyma.08G273500	auxin-responsive protein IAA9(LOC100789251)
38	Glyma.05G229300	auxin-responsive protein IAA27-like(LOC100778529)
39	Glyma.19G221900	auxin-responsive protein IAA20(LOC100783140)
40	Glyma.04G066300	auxin-responsive protein IAA29-like(LOC100802759)

**41**	Glyma.04G247600	IAA-amino acid hydrolase ILR1-like 6(LOC100800794)
**42**	Glyma.06G048400	uncharacterized protein KIAA0930 homolog(LOC100812574)
**43**	Glyma.07G013200	IAA-amino acid hydrolase ILR1-like(LOC100784989)
**44**	Glyma.03G224800	auxin-responsive protein IAA20(LOC100818376)
**45**	Glyma.02G218100	auxin-responsive protein IAA9(LOC100818881)
**46**	Glyma.13G356600	auxin-responsive protein IAA27(LOC100806699)
**47**	Glyma.19G245200	auxin-responsive protein IAA16(LOC100803986)
**48**	Glyma.13G159000	auxin-responsive protein IAA29(LOC100808466)
**49**	Glyma.17G042800	auxin-responsive protein IAA4-like(LOC100527448)
**50**	Glyma.13G361200	AUX/IAA family protein(LOC100500488)
**51**	Glyma.10G138500	auxin-responsive protein IAA30(LOC100781660)
**52**	Glyma.02G007300	auxin-responsive protein IAA30(LOC100784866)
**53**	Glyma.04G226600	IAA-amino acid hydrolase ILR1-like 5(LOC100777587)
**54**	Glyma.15G022300	IAA-amino acid hydrolase ILR1-like 4(LOC100792044)
**55**	Glyma.01G098000	AUX/IAA superfamily protein(LOC100500373)
**56**	Glyma.15G012700	auxin-responsive protein IAA17(LOC100815098)
**57**	Glyma.01G039300	auxin-responsive protein IAA27(LOC100785733)
**58**	Glyma.08G196800	IAA-amino acid hydrolase ILR1-like 4(LOC100789077)
**59**	Glyma.08G196900	IAA-amino acid hydrolase ILR1-like 4-like(LOC100789607)
**60**	Glyma.20G120800	auxin-responsive protein IAA32(LOC100793397)

Table S11. The information of ethylene related genes used in this study

No.	Gene ID	Annotation
**1**	Glyma.07G021000	AP2-like ethylene-responsive transcription factor At1g16060(LOC100807781)
**2**	Glyma.19G262700	ethylene-responsive transcription factor RAP2-2(LOC100810376)
**3**	Glyma.09G240400	AP2-like ethylene-responsive transcription factor At1g16060(LOC100792612)
**4**	Glyma.19G164100	ethylene-response factor C3(LOC100801682)
**5**	Glyma.13G329700	ethylene-responsive transcription factor RAP2-7(LOC100777102)
**6**	Glyma.08G320700	ethylene-responsive transcription factor 3(LOC100795762)
**7**	Glyma.18G252200	ethylene-responsive transcription factor 2(LOC100819961)
**8**	Glyma.19G213300	ethylene response sensor 1(LOC100802569)
**9**	Glyma.17G210500	ethylene-responsive transcription factor SHINE 2(LOC100784305)
**10**	Glyma.10G274600	ethylene-responsive transcription factor ERF118(LOC100306276)
**11**	Glyma.18G252300	ethylene-responsive element binding protein 1(EREBP1)
**12**	Glyma.20G115300	ethylene-responsive transcription factor ERF118(LOC102660769)
**13**	Glyma.01G081100	ethylene-responsive transcription factor ERF118(LOC102664259)
**14**	Glyma.17G131800	ethylene-responsive transcription factor ERF027(LOC100778793)
**15**	Glyma.01G022500	AP2-like ethylene-responsive transcription factor AIL6(LOC100777168)
**16**	Glyma.07G031200	ethylene-responsive transcription factor WIN1(LOC100796962)
**17**	Glyma.18G144700	ethylene-responsive transcription factor ESR2(LOC102661952)
**18**	Glyma.07G055000	ethylene-responsive transcription factor ERF023(LOC100806544)
**19**	Glyma.18G252400	ethylene-responsive transcription factor 1(LOC102663872)
**20**	Glyma.07G114000	ethylene-responsive transcription factor 13(LOC100805648)
**21**	Glyma.08G261500	ethylene-responsive transcription factor ERF118(LOC102667919)
**22**	Glyma.15G221600	ethylene-responsive transcription factor WRI1b(WRI1B)
**23**	Glyma.07G114300	ethylene-responsive transcription factor 13(LOC100804762)
**24**	Glyma.15G025100	ethylene-responsive transcription factor ERF024(LOC100799620)
**25**	Glyma.13G060600	ethylene-responsive transcription factor ERN2(LOC100786711)
**26**	Glyma.03G216700	ethylene response sensor 1(LOC100794417)
**27**	Glyma.10G186800	ethylene-responsive transcription factor 1B(LOC100804481)
**28**	Glyma.17G145300	ethylene-responsive transcription factor 5-like(LOC100816039)
**29**	Glyma.10G186900	ethylene-responsive transcription factor ERF098(LOC100793703)
**30**	Glyma.16G164800	ethylene-responsive transcription factor ABR1(LOC100817436)
**31**	Glyma.13G216900	ethylene-responsive transcription factor ERF086(LOC102664746)
**32**	Glyma.19G253100	ethylene-responsive transcription factor CRF4(LOC102669451)
**33**	Glyma.18G138300	2-methylene-furan-3-one reductase(LOC100780585)
**34**	Glyma.08G227700	ethylene-responsive transcription factor WRI1a(WRI1A)
**35**	Glyma.04G201700	ethylene-responsive transcription factor ERF113(LOC100798671)
**36**	Glyma.03G051300	ethylene-overproduction protein 1(LOC100794758)
**37**	Glyma.07G078600	ethylene-responsive transcription factor ABR1(LOC100799444)
**38**	Glyma.16G007400	AP2-like ethylene-responsive transcription factor AIL1(LOC100809762)
**39**	Glyma.17G169800	ethylene-responsive transcription factor ERF008(LOC100784157)
**40**	Glyma.02G294100	ethylene-responsive transcription factor 3(LOC100803943)

**41**	Glyma.07G113800	ethylene-responsive transcription factor 13(LOC100806359)
**42**	Glyma.18G148000	AP2-like ethylene-responsive transcription factor AIL7(LOC100795280)
**43**	Glyma.06G325300	methylenetetrahydrofolate reductase 2(LOC100797489)
**44**	Glyma.13G236500	ethylene-responsive element binding factor 4(LOC100170742)
**45**	Glyma.13G236600	ethylene-responsive transcription factor 12(LOC100798369)
**46**	Glyma.02G039300	ethylene-responsive transcription factor ESR1(ESR1)
**47**	Glyma.15G079200	ethylene-response factor C3(LOC112999553)
**48**	Glyma.07G025800	ethylene-responsive transcription factor ERF003-like(LOC100777851)
**49**	Glyma.04G201900	ethylene-responsive transcription factor ERF114(LOC100799190)
**50**	Glyma.16G154100	ethylene-responsive transcription factor ERN1(LOC100792432)
**51**	Glyma.06G105000	ethylene-responsive transcription factor RAP2-4(LOC100800478)
**52**	Glyma.11G036500	ethylene-responsive transcription factor 2(LOC100819649)
**53**	Glyma.17G194100	ethylene-responsive transcription factor TINY(LOC100816404)
**54**	Glyma.20G155200	ethylene-responsive transcription factor ERF027(LOC100776717)
**55**	Glyma.11G036400	ethylene-responsive transcription factor 5-like protein(LOC100800788)
**56**	Glyma.16G046300	ethylene-responsive transcription factor LEP(LOC100792421)
**57**	Glyma.17G145400	ethylene-responsive transcription factor 1A(LOC100817097)
**58**	Glyma.17G062600	AP2-like ethylene-responsive transcription factor AIL5(LOC100803558)
**59**	Glyma.09G194800	ethylene-responsive transcription factor TINY(LOC100784134)
**60**	Glyma.15G077000	ethylene-responsive transcription factor 12(LOC100802655)
**61**	Glyma.14G106200	ethylene-responsive transcription factor ERF017(LOC112999363)
**62**	Glyma.19G008700	2-methylene-furan-3-one reductase(LOC100793908)
**63**	Glyma.19G008600	2-methylene-furan-3-one reductase(LOC100792857)
**64**	Glyma.08G257300	ethylene-responsive transcription factor ERF113(LOC100796624)
**65**	Glyma.16G012600	ethylene responsive protein(EREB)
**66**	Glyma.02G229400	ethylene-overproduction protein 1(LOC100800568)
**67**	Glyma.19G008500	2-methylene-furan-3-one reductase(LOC100791274)
**68**	Glyma.13G123100	ethylene-responsive transcription factor 1-like protein(LOC100784784)
**69**	Glyma.15G018400	ethylene-responsive transcription factor ERF036(LOC100787471)
**70**	Glyma.12G117000	ethylene-responsive transcription factor 9(LOC100775465)
**71**	Glyma.01G224100	ethylene-responsive transcription factor CRF2(LOC100783772)
**72**	Glyma.10G116600	AP2-like ethylene-responsive transcription factor TOE3(LOC100783626)
**73**	Glyma.09G072000	ethylene-responsive transcription factor ERF017(LOC100802508)
**74**	Glyma.12G073300	ethylene-responsive transcription factor RAP2-7(LOC100788303)
**75**	Glyma.15G004200	ethylene-responsive transcription factor ERF003(LOC100805499)
**76**	Glyma.15G077100	ethylene-responsive transcription factor 4(LOC100803173)
**77**	Glyma.07G250100	ethylene-responsive transcription factor ERF086(LOC100812047)
**78**	Glyma.14G111600	ethylene-responsive transcription factor ERF003(LOC100807612)
**79**	Glyma.05G157400	ethylene-responsive transcription factor ERN3(LOC100803832)
**80**	Glyma.16G148700	ethylene-responsive transcription factor LEP(LOC100809600)

**81**	Glyma.02G132500	ethylene-responsive transcription factor ERF109(LOC100794381)
**82**	Glyma.05G108600	AP2-like ethylene-responsive transcription factor ANT(LOC100787611)
**83**	Glyma.05G049800	ethylene-responsive transcription factor ERF026(LOC100793589)
**84**	Glyma.11G045800	AP2-like ethylene-responsive transcription factor ANT(LOC100794433)
**85**	Glyma.07G044300	ethylene-responsive transcription factor 7(ERF7)
**86**	Glyma.20G087000	ethylene receptor 2(LOC100802948)
**87**	Glyma.18G059700	ethylene-overproduction protein 1(LOC100790527)
**88**	Glyma.10G219000	ethylene-responsive transcription factor LEP(LOC100816937)
**89**	Glyma.05G015900	ethylene-response factor C3(LOC100807374)
**90**	Glyma.08G115000	ethylene-responsive transcription factor ERN3(LOC100809046)
**91**	Glyma.20G195900	putative ethylene-responsive transcription factor(LOC100306125)
**92**	Glyma.17G254600	ethylene-responsive transcription factor ERF043(LOC100776824)
**93**	Glyma.05G186700	ethylene-responsive transcription factor ABR1(LOC100817546)
**94**	Glyma.17G240100	ethylene-responsive transcription factor RAP2-1(LOC100810839)
**95**	Glyma.09G053000	ethylene-responsive transcription factor ERF098(LOC100807295)
**96**	Glyma.04G057700	ethylene-responsive transcription factor ERF016(LOC100808434)
**97**	Glyma.13G274100	ethylene-responsive transcription factor 4(LOC100813652)
**98**	Glyma.10G171400	AP2-like ethylene-responsive transcription factor BBM(LOC100808723)
**99**	Glyma.11G167200	ethylene-overproduction protein 1(LOC100800616)
**100**	Glyma.03G111700	ethylene-responsive transcription factor 13(LOC100793366)
**101**	Glyma.03G159800	ethylene-responsive transcription factor 12(LOC100816599)
**102**	Glyma.02G067600	ethylene-responsive transcription factor LEP-like(LOC100815668)
**103**	Glyma.13G166700	ethylene-responsive transcription factor WIN1(LOC100814893)
**104**	Glyma.04G047900	AP2-like ethylene-responsive transcription factor ANT(LOC100787223)
**105**	Glyma.07G038200	AP2-like ethylene-responsive transcription factor AIL1(LOC100805489)
**106**	Glyma.15G152000	ethylene-responsive transcription factor RAP2-3(LOC100779626)
**107**	Glyma.13G298600	ethylene-responsive transcription factor ERF053(LOC100809887)
**108**	Glyma.02G185200	AP2-like ethylene-responsive transcription factor At2g41710(LOC100795084)
**109**	Glyma.08G145300	ethylene-responsive transcription factor ERF112(LOC100792946)
**110**	Glyma.13G103500	enhanced ethylene response protein 5(LOC100781018)
**111**	Glyma.03G140800	AP2-like ethylene-responsive transcription factor BBM(LOC102664631)
**112**	Glyma.17G219700	ethylene-responsive transcription factor ERF017(LOC102665533)
**113**	Glyma.13G040400	ethylene-responsive transcription factor CRF3(LOC100801199)
**114**	Glyma.04G067200	ethylene-responsive transcription factor SHINE 2(LOC100806821)
**115**	Glyma.19G248900	ethylene-response factor C3(LOC100787732)
**116**	Glyma.15G044400	ethylene-responsive transcription factor RAP2-7(LOC100796271)
**117**	Glyma.10G058300	ethylene-insensitive protein 2(LOC100781309)
**118**	Glyma.09G199800	AP2-like ethylene-responsive transcription factor AIL6(LOC100775390)
**119**	Glyma.13G088100	ethylene-responsive transcription factor RAP2-4(LOC100802961)
**120**	Glyma.02G087400	AP2-like ethylene-responsive transcription factor TOE3(LOC100776138)
**121**	Glyma.17G024300	ethylene-responsive transcription factor ERF086(LOC100793333)
**122**	Glyma.10G239300	ethylene-responsive transcription factor ERF027(LOC100813560)
**123**	Glyma.13G151900	ethylene-responsive transcription factor ERF024(LOC100782443)

**124**	Glyma.12G241700	ethylene receptor(LOC100795340)
**125**	Glyma.06G221800	ethylene-responsive transcription factor WIN1(LOC100819868)
**126**	Glyma.17G070800	AP2-like ethylene-responsive transcription factor At1g16060(LOC100790871)
**127**	Glyma.13G112400	ethylene-responsive transcription factor ERF017(LOC100804384)
**128**	Glyma.10G007100	ethylene-responsive transcription factor ERF098(LOC100814296)
**129**	Glyma.10G007000	ethylene-response factor C3(LOC100785936)
**130**	Glyma.04G256100	methylenetetrahydrofolate reductase 2(LOC100782073)
**131**	Glyma.09G242600	ethylene-responsive transcription factor 1(LOC100806588)
**132**	Glyma.06G049200	AP2-like ethylene-responsive transcription factor ANT(LOC100802454)
**133**	Glyma.17G143900	ethylene-responsive transcription factor ERF039(LOC100812293)
**134**	Glyma.16G147500	ethylene-responsive transcription factor ERF113(LOC100806383)
**135**	Glyma.12G182400	ethylene-responsive transcription factor ERF024(LOC100816109)
**136**	Glyma.06G290000	ethylene-responsive transcription factor 9(LOC100801924)
**137**	Glyma.03G116700	ethylene-responsive transcription factor ERF039-like(LOC100801129)
**138**	Glyma.20G196400	ethylene-responsive transcription factor 2(LOC102664672)
**139**	Glyma.06G020700	24-methylenesterol C-methyltransferase 2-2(SMT2-2)
**140**	Glyma.13G122500	ethylene-responsive transcription factor ERF096(LOC100810414)
**141**	Glyma.06G068800	ethylene-responsive transcription factor SHINE 2(LOC100794649)
**142**	Glyma.10G223200	ethylene-responsive transcription factor ERF110(LOC100777915)
**143**	Glyma.13G122700	ethylene-response factor C3(LOC100811486)
**144**	Glyma.10G188500	ethylene receptor(ETR2)
**145**	Glyma.13G096900	AP2-like ethylene-responsive transcription factor AIL5(LOC100797828)
**146**	Glyma.16G040000	ethylene-responsive transcription factor ERN1(LOC100782886)
**147**	Glyma.13G122800	ethylene-response factor C3(LOC100812017)
**148**	Glyma.08G297000	AP2-like ethylene-responsive transcription factor At1g79700(LOC100799621)
**149**	Glyma.05G063500	ethylene-responsive transcription factor 5(LOC100800453)
**150**	Glyma.04G217400	ethylene-responsive transcription factor ABR1(LOC100793215)
**151**	Glyma.02G072800	ethylene-responsive transcription factor ERN1(LOC100527497)
**152**	Glyma.13G122900	ethylene-responsive transcription factor 1B(LOC100812569)
**153**	Glyma.15G159100	ethylene-responsive transcription factor ERF098(LOC100796977)
**154**	Glyma.17G114500	ethylene-responsive transcription factor WIN1(LOC100787517)
**155**	Glyma.05G063600	ethylene-responsive transcription factor 1A(LOC100803653)
**156**	Glyma.15G159200	ethylene-responsive transcription factor ERF098(LOC100797513)
**157**	Glyma.14G171500	ethylene-responsive transcription factor RAP2-4(LOC100781774)
**158**	Glyma.17G055500	enhanced ethylene response protein 5(LOC100795595)
**159**	Glyma.12G226600	ethylene-responsive transcription factor 6(ERF6)
**160**	Glyma.15G180000	ethylene-responsive transcription factor ERF017(LOC100813688)
**161**	Glyma.18G018200	ethylene-responsive transcription factor ERF060(LOC106796800)
**162**	Glyma.03G112800	ethylene-responsive transcription factor 13(LOC102661516)
**163**	Glyma.06G058400	ethylene-responsive transcription factor TINY(LOC100817386)

**164**	Glyma.10G066900	ethylene-responsive transcription factor ERF024(LOC100811614)
**165**	Glyma.08G215700	ethylene-responsive transcription factor ERF084(LOC102667667)
**166**	Glyma.07G027000	ethylene-responsive transcription factor ERF084(LOC102660200)
**167**	Glyma.20G172800	ethylene-responsive transcription factor LEP(LOC100809161)
**168**	Glyma.11G019000	ethylene-responsive transcription factor CRF1(LOC100779905)
**169**	Glyma.05G092800	ethylene-responsive transcription factor ERF010(LOC100789373)
**170**	Glyma.03G112000	ethylene-responsive transcription factor 2(LOC100792115)
**171**	Glyma.12G182200	ethylene-responsive transcription factor ERF027(LOC100815039)
**172**	Glyma.11G014800	ethylene-responsive transcription factor ERF020(LOC100816257)
**173**	Glyma.01G225000	ethylene-responsive transcription factor ERF061(LOC100785364)
**174**	Glyma.03G136100	AP2-like ethylene-responsive transcription factor At2g41710(LOC100814117)
**175**	Glyma.08G220800	AP2-like ethylene-responsive transcription factor(LOC100819898)
**176**	Glyma.03G112100	ethylene-responsive transcription factor 2(LOC100793895)
**177**	Glyma.14G020100	ethylene-responsive transcription factor 3(LOC100820249)
**178**	Glyma.03G258200	ethylene-responsive transcription factor ERF023(LOC100819971)
**179**	Glyma.11G239200	ethylene-responsive transcription factor ERF060-like(LOC100815162)
**180**	Glyma.19G113100	ethylene-responsive transcription factor ERN1(ERN)
**181**	Glyma.18G244600	AP2-like ethylene-responsive transcription factor BBM1(LOC100797584)
**182**	Glyma.01G195900	AP2-like ethylene-responsive transcription factor ANT(LOC100812275)
**183**	Glyma.03G112400	ethylene-responsive transcription factor 1(LOC100793710)
**184**	Glyma.04G154900	AP2-like ethylene-responsive transcription factor At1g16060(LOC100795869)
**185**	Glyma.20G070000	ethylene-responsive transcription factor ERF105(LOC100795866)
**186**	Glyma.20G202200	ethylene receptor 2(LOC100787425)
**187**	Glyma.15G008600	ethylene-responsive transcription factor WIN1(LOC100778381)
**188**	Glyma.05G200100	ethylene-responsive transcription factor ERF114(LOC100793410)
**189**	Glyma.05G179900	ethylene-responsive transcription factor ERF060(LOC100797648)
**190**	Glyma.06G148400	ethylene-responsive transcription factor ERF110(LOC100796247)
**191**	Glyma.04G147500	ethylene-responsive transcription factor WIN1(LOC100779921)
**192**	Glyma.08G137600	ethylene-responsive transcription factor(LOC100818839)
**193**	Glyma.05G214400	ethylene-responsive transcription factor CRF2(LOC100813992)
**194**	Glyma.18G159900	ethylene-responsive transcription factor-like protein At4g13040(LOC100786819)
**195**	Glyma.19G104200	AP2-like ethylene-responsive transcription factor BBM1(LOC106797385)
**196**	Glyma.08G020900	ethylene-responsive transcription factor CRF2(LOC100776453)
**197**	Glyma.08G211600	ethylene-responsive transcription factor WIN1(LOC100785898)
**198**	Glyma.14G070000	ethylene-responsive transcription factor ERF043(LOC100794497)
**199**	Glyma.11G014200	ethylene-responsive transcription factor ERF021(LOC100815719)
**200**	Glyma.10G187000	ethylene-responsive transcription factor ERF096(LOC100794227)
**201**	Glyma.13G233800	ethylene-response factor C3(LOC100820226)
**202**	Glyma.10G118900	ethylene-responsive transcription factor ERF105(LOC100816416)
**203**	Glyma.06G325100	carboxymethylenebutenolidase homolog(LOC100796954)
**204**	Glyma.20G031000	ethylene-responsive transcription factor ERN2(LOC100793940)
**205**	Glyma.19G138000	AP2-like ethylene-responsive transcription factor At2g41710(LOC100786651)
**206**	Glyma.11G053600	ethylene-responsive transcription factor ERF011(LOC100783682)

**207**	Glyma.14G050100	ethylene-responsive transcription factor 3(LOC100795557)
**208**	Glyma.13G145100	ethylene-insensitive protein 2(LOC100801190)
**209**	Glyma.03G181400	ethylene-insensitive protein 2(LOC100813034)
**210**	Glyma.01G232000	ethylene-responsive transcription factor ERF020(LOC100803550)
**211**	Glyma.06G295300	ethylene-responsive transcription factor ERF054(LOC100811328)
**212**	Glyma.06G163700	ethylene-responsive transcription factor ERF114(LOC100787091)
**213**	Glyma.12G056300	AP2-like ethylene-responsive transcription factor PLT2(LOC100788471)
**214**	Glyma.10G016500	ethylene-responsive transcription factor RAP2-2-like(LOC100780763)
**215**	Glyma.20G197000	ethylene-responsive transcription factor ERF062(LOC100820013)
**216**	Glyma.07G091100	ethylene-responsive transcription factor ERF117(LOC102661579)
**217**	Glyma.14G123900	ethylene-responsive transcription factor CRF1(LOC100805277)
**218**	Glyma.19G192400	ethylene-responsive transcription factor ERF024(LOC100813781)
**219**	Glyma.01G123900	ethylene-overproduction protein 1(LOC100799641)
**220**	Glyma.14G197100	ethylene-overproduction protein 1(LOC100799963)
**221**	Glyma.02G006200	ethylene-responsive transcription factor 1B(LOC100780576)
**222**	Glyma.01G025400	ethylene-responsive transcription factor ESR2(LOC102669715)
**223**	Glyma.01G147600	ethylene-responsive transcription factor TINY(LOC100804801)
**224**	Glyma.02G006300	ethylene-responsive transcription factor ERF096(LOC100781117)
**225**	Glyma.02G080200	ethylene-responsive transcription factor ABR1(LOC100804843)
**226**	Glyma.01G074200	ethylene-responsive transcription factor ERF034(LOC100792964)
**227**	Glyma.08G202300	ethylene-responsive transcription factor ERF036(LOC100801780)
**228**	Glyma.17G158300	AP2-like ethylene-responsive transcription factor ANT(LOC100792451)
**229**	Glyma.18G262800	ethylene-responsive transcription factor ERN1(LOC100796159)
**230**	Glyma.14G089200	AP2-like ethylene-responsive transcription factor CRL5(LOC100808138)
**231**	Glyma.18G125200	AP2-like ethylene-responsive transcription factor At1g16060(LOC100798483)
**232**	Glyma.01G231200	ethylene-responsive transcription factor ERF021(LOC100801245)
**233**	Glyma.01G231000	ethylene-responsive transcription factor ERF022(LOC100788567)
**234**	Glyma.13G369400	ethylene-responsive transcription factor ERF003(LOC100812021)
**235**	Glyma.07G110000	ethylene-responsive transcription factor ERF034(LOC100776436)
**236**	Glyma.06G236400	ethylene-responsive transcription factor 4(LOC100786709)
**237**	Glyma.19G256800	ethylene-responsive transcription factor ERF023(LOC100802036)
**238**	Glyma.08G348300	ethylene-responsive transcription factor-like protein At4g13040(LOC100306701)
**239**	Glyma.03G255500	ethylene-responsive transcription factor CRF6(LOC100814120)
**240**	Glyma.10G036300	ethylene-response factor C3(LOC100782036)
**241**	Glyma.08G040900	ethylene-responsive transcription factor CRF3(LOC102661155)
**242**	Glyma.19G163700	ethylene-responsive transcription factor ERF095(LOC100800069)
**243**	Glyma.16G023800	ethylene-responsive transcription factor ERF023(LOC100802845)
**244**	Glyma.10G036200	ethylene-responsive transcription factor ERF096(LOC100781487)
**245**	Glyma.15G095600	ethylene-responsive transcription factor ERF086(LOC102661302)
**246**	Glyma.09G002600	ethylene receptor(LOC100795799)

**247**	Glyma.18G091600	ethylene-responsive transcription factor 7(LOC100782025)
**248**	Glyma.04G041200	ethylene-responsive transcription factor RAP2-1(LOC100801528)
**249**	Glyma.02G207100	AP2-like ethylene-responsive transcription factor At1g16060(LOC100793339)
**250**	Glyma.12G110400	ethylene-responsive transcription factor ERF054(LOC100801882)
**251**	Glyma.20G203700	ethylene-responsive transcription factor 1B(LOC100790598)
**252**	Glyma.08G035000	ethylene-responsive transcription factor TINY(LOC100778201)
**253**	Glyma.10G036700	ethylene-response factor C3(LOC100780775)
**254**	Glyma.20G203600	ethylene-responsive transcription factor ERF098(LOC100776376)
**255**	Glyma.10G036600	ethylene-responsive transcription factor ERF098-like protein(LOC100779173)
**256**	Glyma.08G279000	AP2-like ethylene-responsive transcription factor AIL6(LOC100814405)
**257**	Glyma.20G203500	ethylene-responsive transcription factor ERF096(LOC100775830)
**258**	Glyma.19G026000	ethylene-responsive transcription factor ERN2(LOC100790398)
**259**	Glyma.03G162700	ethylene-response factor C3(LOC100779524)
**260**	Glyma.20G168500	ethylene-responsive transcription factor ERF110(LOC100802242)
**261**	Glyma.10G193400	ethylene-responsive transcription factor ERF062(LOC100819431)
**262**	Glyma.03G162600	ethylene-responsive transcription factor ERF098(LOC100778998)
**263**	Glyma.19G163900	ethylene-response factor C3(LOC100800600)
**264**	Glyma.09G041500	ethylene-responsive transcription factor RAP2-3-like(ERF5)
**265**	Glyma.16G047600	ethylene-responsive transcription factor ERF113(LOC100816370)
**266**	Glyma.01G216200	ethylene-responsive transcription factor ERF027(LOC100811020)
**267**	Glyma.03G162500	ethylene-responsive transcription factor 15-like(LOC100818907)
