# Supplementary figures and images for: Genetic and transcriptome analyses reveal the candidate genes and pathways involved in the inactive shade-avoidance response enabling high-density planting of soybean
Source: Front Plant Sci. 2022 Aug 3;13:973643. doi: 10.3389/fpls.2022.973643 (PMC9382032; doi:10.3389/fpls.2022.973643)

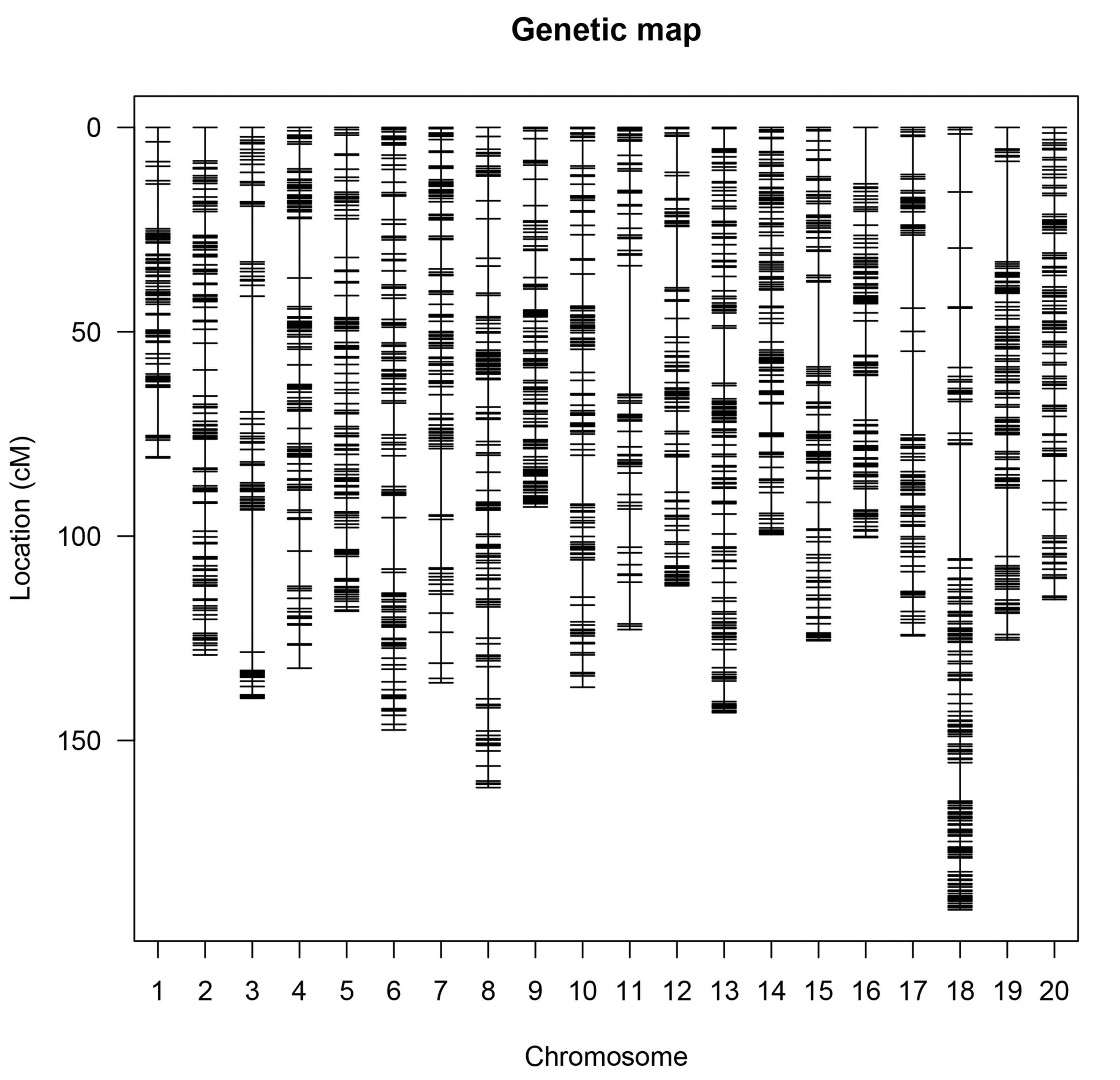

Supplement: SUPPLEMENTARY FIGURE 1 — The genetic map of the1712 recombinant inbred line (RIL) population. The genetic map was constructed used the qtl package of R language. [file Image_1.jpg]
